# High-mobility group box 1 increases platelet surface P2Y_12_ and platelet activation in sickle cell disease

**DOI:** 10.1172/jci.insight.174575

**Published:** 2024-03-08

**Authors:** Deirdre Nolfi-Donegan, Gowtham K. Annarapu, Claudette St. Croix, Michael Calderon, Cheryl A. Hillery, Sruti Shiva

**Affiliations:** 1 Department of Pediatrics, Division of Hematology/Oncology, Children’s Hospital of Pittsburgh, Pittsburgh, Pennsylvania, USA.; 2 University of Pittsburgh School of Medicine,; 3 Pittsburgh Heart, Lung, Blood Vascular Medicine Institute,; 4 Center for Biologic Imaging,; 5 Center for Metabolism & Mitochondrial Medicine, and; 6 Department of Pharmacology & Chemical Biology, University of Pittsburgh, Pittsburgh, Pennsylvania, USA.

**Keywords:** Cell biology, Hematology, Platelets, Thrombosis

## Abstract

Thrombosis and inflammation are intimately linked and synergistically contribute to the pathogenesis of numerous thromboinflammatory diseases, including sickle cell disease (SCD). While platelets are central to thrombogenesis and inflammation, the molecular mechanisms of crosstalk between the 2 remain elusive. High-mobility group box 1 (HMGB1) regulates inflammation and stimulates platelet activation through Toll-like receptor 4. However, it remains unclear whether HMGB1 modulates other thrombotic agonists to regulate platelet activation. Herein, using human platelets, we demonstrate that HMGB1 significantly enhanced ADP-mediated platelet activation. Furthermore, inhibition of the purinergic receptor P2Y_12_ attenuated HMGB1-dependent platelet activation. Mechanistically, we show that HMGB1 stimulated ADP secretion, while concomitantly increasing P2Y_12_ levels at the platelet membrane. We show that in SCD patients, increased plasma HMGB1 levels were associated with heightened platelet activation and surface P2Y_12_ expression. Treatment of healthy platelets with plasma from SCD patients enhanced platelet activation and surface P2Y_12_, and increased sensitivity to ADP-mediated activation, and these effects were linked to plasma HMGB1. We conclude that HMGB1-mediated platelet activation involves ADP-dependent P2Y_12_ signaling, and HMGB1 primes platelets for ADP signaling. This complementary agonism between ADP and HMGB1 furthers the understanding of thromboinflammatory signaling in conditions such as SCD, and provides insight for therapeutic P2Y_12_ inhibition.

## Abstract 2

HMGB1 increases platelet surface P2Y12 and promotes platelet ADP release, thereby enhancing ADP-mediated platelet activation in healthy subjects and individuals with sickle cell disease.

## Graphical abstract

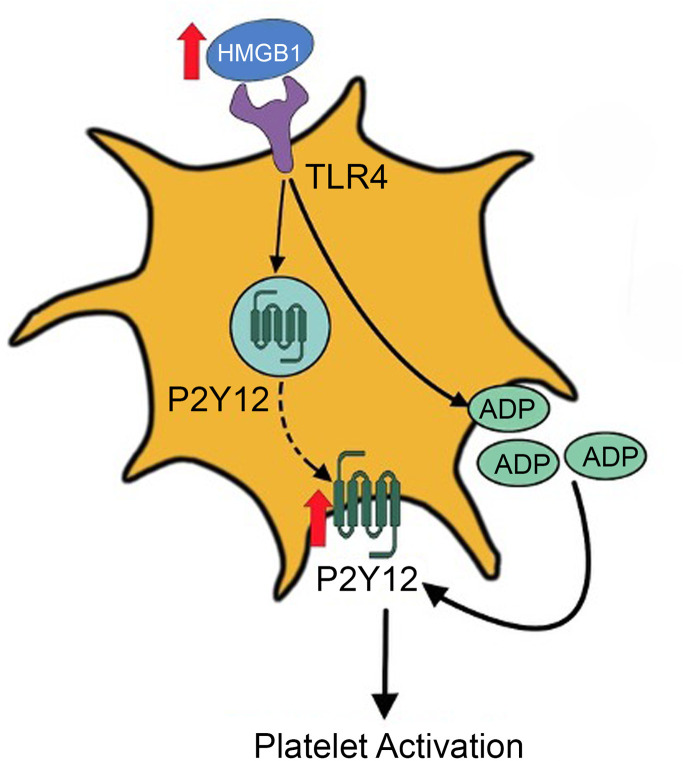

## Introduction

Thrombosis and inflammation are intimately linked (1–3), and converge at the level of the platelet. While platelets are well established as integral players in thrombus formation, accumulating data demonstrate that the platelet is also a sentinel for inflammatory signaling (1–4). Platelets identify inflammatory stimuli through pattern recognition receptors, including Toll-like receptors (TLRs), which initiate responses that propagate inflammatory signaling (5). While many of these platelet responses, such as the secretion of cytokines, neutrophil binding to instigate neutrophil extracellular trap (NET) formation, and tethering of leukocytes to the endothelium, require platelet activation (6, 7), the mechanisms of crosstalk between inflammatory and thrombotic stimuli that potentiate platelet activation remain unclear.

High-mobility group box 1 (HMGB1) is a highly conserved 215–amino acid DNA-binding protein that functions as a mediator of sterile inflammation (8). HMGB1 is released into the circulation by damaged tissue or actively secreted by immunogenic cells (9). Platelets also store HMGB1 and release it or present it on their surface upon activation (9–11). Surface expression of HMGB1 enables platelet inflammatory signaling through the formation of platelet-monocyte aggregates or through interactions with neutrophils to form NETs (12), while HMGB1 interaction with TLR4 on the platelet surface activates the NOD-, LRR-, and pyrin domain–containing protein 3 (NLRP3) inflammasome (13) and stimulates the production of platelet-derived microparticles (11). On a mechanistic level, HMGB1 is redox regulated, and depending on its oxidation state, stimulates platelet activation predominantly through TLR4 or the receptor for advanced glycation end products (RAGE) (12, 14–17). While these studies demonstrate that HMGB1 can directly stimulate platelet activation, it is unclear whether HMGB1 enhances the effect of other platelet agonists to potentiate platelet activation during inflammation.

Adenosine diphosphate (ADP), stored in platelet dense granules, is a relatively weak platelet agonist. Through activation of purinergic receptors, predominantly P2Y_12_, ADP mediates increases in intracellular calcium and activation of integrin αIIbβ3, along with platelet shape change and aggregation (4). Antagonists of P2Y_12_ are potent inhibitors of platelet thrombotic function and are thus utilized clinically as prophylaxis in cardiovascular disease and embolic stroke (18). Notably, several studies have implicated P2Y_12_ receptors in modulating inflammatory responses. For example, mice or patients receiving P2Y_12_ antagonists show decreased levels of inflammatory markers, including circulating CD40 ligand (19, 20), IL-6 (21), C-reactive protein (22, 23), and platelet-leukocyte aggregates (24). However, it remains unknown whether inflammatory signaling molecules such as HMGB1 directly regulate P2Y_12_ or crosstalk with ADP-mediated signaling.

Sickle cell disease (SCD) is the most common inherited hemoglobinopathy, and thromboinflammatory signaling plays a significant role in the pathogenesis of vaso-occlusive crisis as well as the long-term sequelae of SCD (25). SCD patients show increased thrombotic tendency, including increased risk of childhood ischemic stroke (26, 27) and venous thromboembolism (3, 28, 29). SCD patients exhibit chronic hemostatic activation (25), including enhanced tissue factor activity (30, 31), increased fibrin and thrombin generation (32, 33), and increased platelet activation at baseline and in crisis (34–37), which likely contribute to the thrombotic complications observed in this population. Importantly, SCD patients also exhibit hallmarks of chronic sterile inflammation resulting from hemolysis and repeated ischemia-reperfusion injury to tissues and vasculature (38); these include leukocytosis, leukocyte activation (39), excess proinflammatory cytokines (40), endothelial activation (38), and NET formation (41).

Circulating levels of both ADP and HMGB1 are significantly increased in patients with SCD (8, 42), contributing to the thromboinflammatory milieu. Murine models of SCD have demonstrated that ADP released from hemolytic erythrocytes contributes to platelet activation (43), while HMGB1 signaling has been shown to upregulate the platelet inflammasome (13, 17). While large randomized control trials of P2Y_12_ antagonists in SCD patients have shown mixed results in terms of preventing vaso-occlusive complications and have not focused on molecular markers of thrombosis or inflammation (44–46), these antagonists have demonstrated an attenuation of inflammatory signaling in smaller human studies, and in preclinical murine models of SCD (47–49).

Herein, we tested whether HMGB1 synergizes with the ADP purinergic signaling axis to modulate platelet activation. We demonstrate that HMGB1 potentiates platelet activation mediated by ADP. Mechanistically, HMGB1, through the activation of TLR4, increases P2Y_12_ at the platelet surface via dynein-dependent transport. In individuals with SCD, we demonstrate that P2Y_12_ is increased on the platelet surface, and that elevated plasma HMGB1 contributes to ADP-dependent platelet activation. The implications of these studies will be discussed in the context of thromboinflammatory signaling and therapeutic inhibition of P2Y_12_ receptors.

## Results

### HMGB1 enhances ADP-dependent platelet activation.

**Figure 1:**
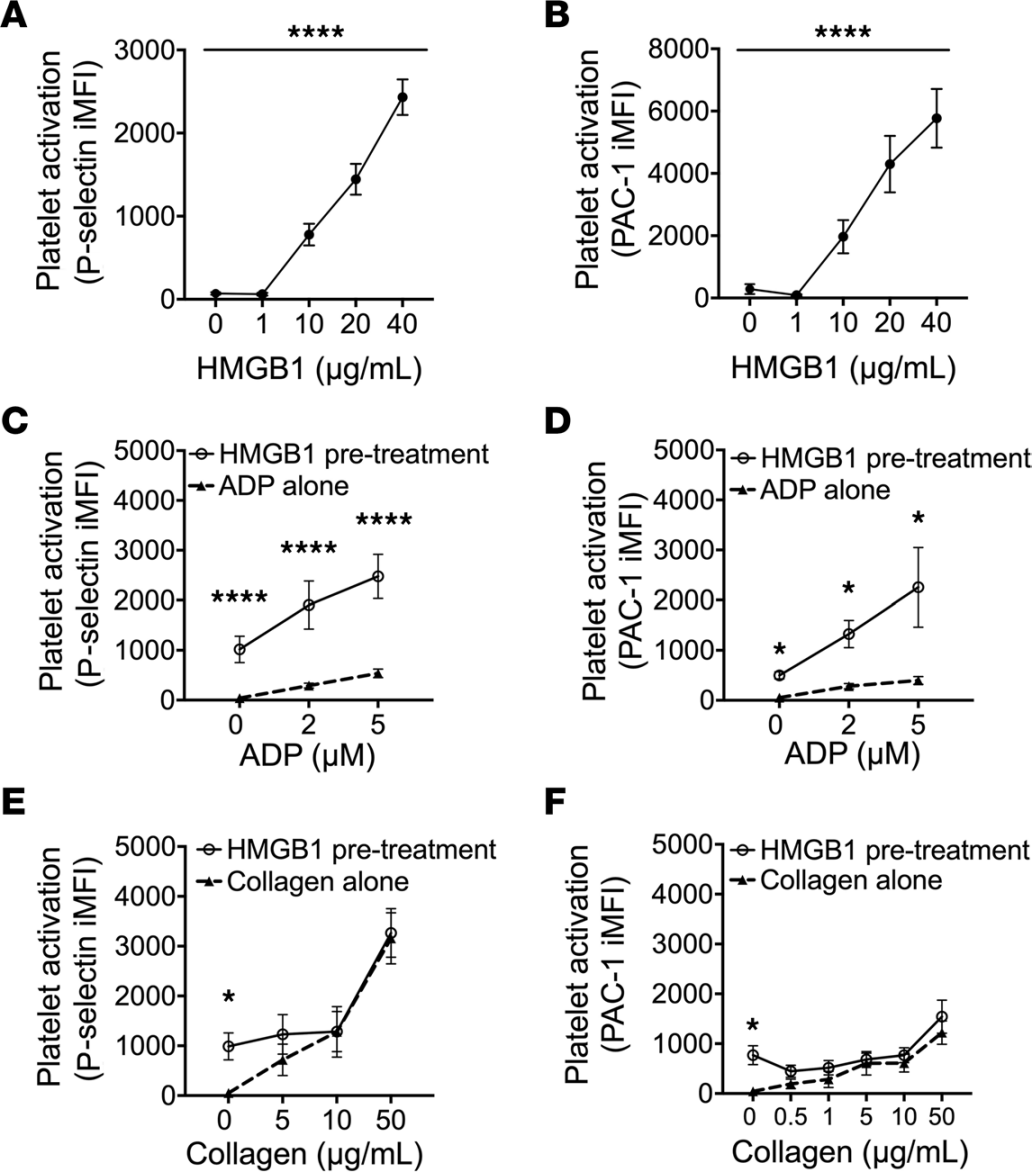
HMGB1 sensitizes platelets to ADP-dependent platelet activation. (**A**) Surface P-selectin and (**B**) integrin αIIbβ3 activation–dependent conformational change (PAC-1) were measured in isolated human platelets in response to treatment with recombinant HMGB1 (0–40 μg/mL) (1-way ANOVA with Tukey’s post hoc test, *n* ≥ 4). (**C** and **E**) Surface P-selectin and (**D** and **F**) activated integrin αIIbβ3 after treatment with (**C** and **D**) ADP alone (dashed lines) compared to ADP after pretreatment with HMGB1 (10 μg/mL; solid lines) and with (**E** and **F**) collagen alone (dashed lines) compared to collagen pretreated with HMGB1 (solid lines). *n* ≥ 4. Comparisons were analyzed by 2-way ANOVA with Bonferroni’s correction. All data are mean ± SEM. *P ≤ 0.05; ****P ≤ 0.0001.

To determine whether HMGB1 synergizes with the ADP-dependent purinergic platelet activation pathway, we characterized HMGB1- and ADP-dependent platelet activation independently and in combination. Isolated human platelets were treated with recombinant HMGB1 (0–40 μg/mL) and platelet activation was measured. Measurement of surface P-selectin expression (Figure 1A) and the activation-dependent conformation of integrin αIIbβ3 (Figure 1B) showed a concentration-dependent increase, confirming prior studies showing that HMGB1 activates human platelets. Notably, the recombinant HMGB1 used in these studies was partially oxidized (disulfide bridge between Cys23 and Cys25, while Cys106 remains reduced). The fully oxidized (all 3 thiols oxidized) or fully reduced (all 3 thiols reduced) forms of HMGB1 mediated much less platelet activation compared with the partially oxidized form (Supplemental Figure 1, A–C; supplemental material available online with this article; https://doi.org/10.1172/jci.insight.174575DS1), consistent with prior studies demonstrating that the partially oxidized form mediates maximal platelet activation (9, 15–17, 50). Thus, we used the partially oxidized HMGB1 for further studies.

To determine whether HMGB1 modulates ADP-stimulated activation, platelets were pretreated with HMGB1 (10 μg/mL) prior to incubation with ADP (0–5 μM). While ADP alone induced platelet activation in a concentration-dependent manner, HMGB1 pretreatment significantly potentiated platelet activation measured by both surface P-selectin expression (Figure 1C) and integrin αIIbβ3 (Figure 1D). In contrast, HMGB1 pretreatment did not significantly change collagen-induced platelet activation (Figure 1, E and F). These data demonstrate that not only does HMGB1 independently stimulate platelet activation, it also enhances ADP-dependent platelet activation to a level greater than either agonist alone.

### Inhibition of P2Y_12_ attenuates HMGB1-dependent platelet activation in vitro and in vivo.

**Figure 2:**
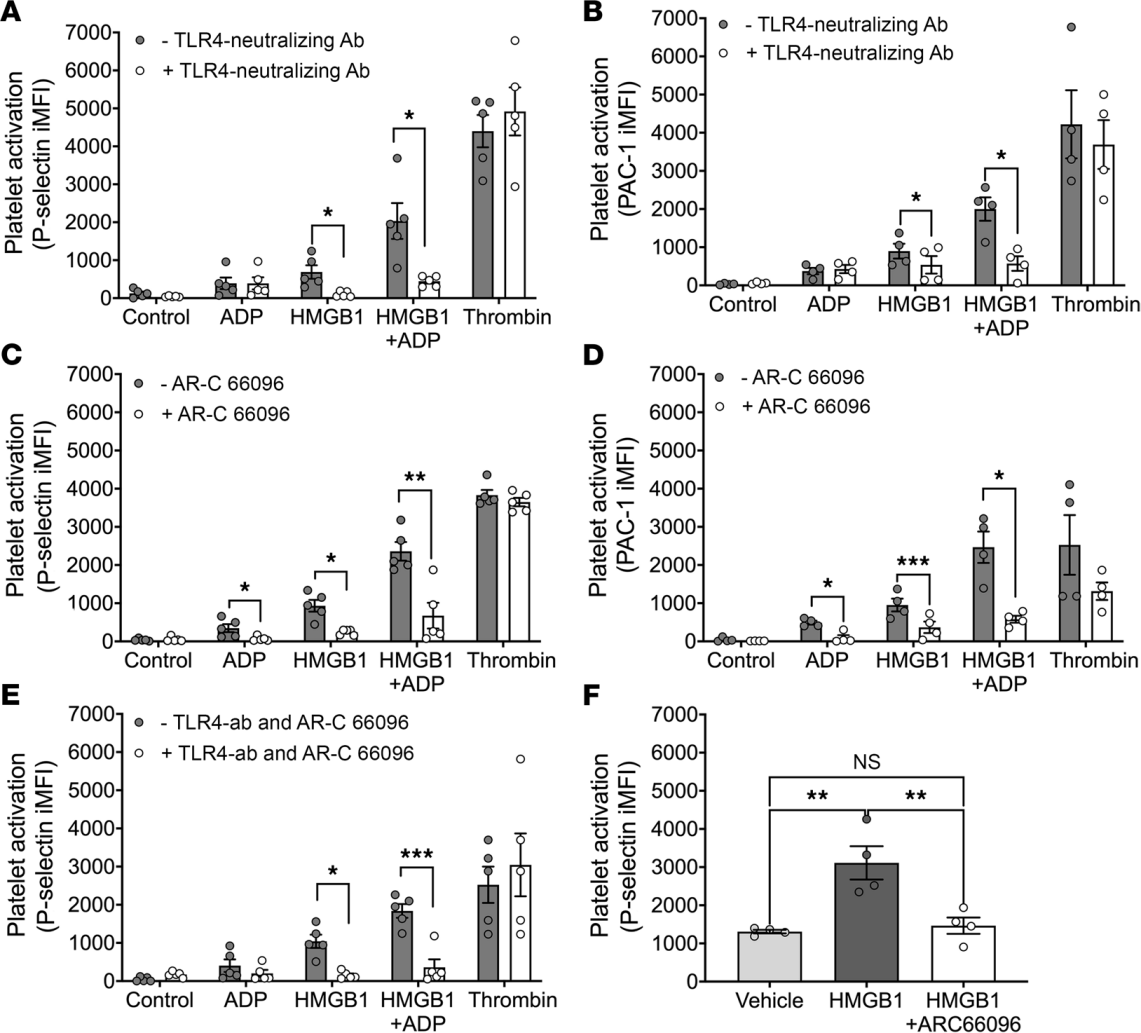
HMGB1 activates platelets via TLR4- and P2Y_12_-dependent signaling. (**A**) Surface P-selectin and (**B**) activated integrin αIIbβ3 (PAC-1) in isolated human platelets stimulated with ADP (5 μM), HMGB1 (10 μg/mL), HMGB1 plus ADP, or thrombin control (0.1 U/mL) alone, and after pretreatment with (**A** and **B**) anti-TLR4 neutralizing antibody (1 μg/mL, *n* ≥ 4); (**C** and **D**) AR-C 66096 (P2Y_12_ inhibitor; 1 μM, *n* ≥ 4); or (**E**) with AR-C 66096 and anti-TLR4 neutralizing antibody (1 μg/mL, *n* = 5). Data in **A**–**E** are mean ± SEM and comparisons were analyzed by paired, 2-tailed *t* test. (**F**) P-selectin on platelets from C57BL/6J mice treated with vehicle control (vehicle), HMGB1 alone (1 μg/g), or pretreated with AR-C 66096 (3 μg/g) before HMGB1 treatment. *n* = 4. Data are mean ± SEM and were analyzed by 1-way ANOVA with Tukey’s post hoc test for column-to-column comparisons. NS, not significant. *P ≤ 0.05; **P ≤ 0.01; ***P ≤ 0.001.

To determine whether the purinergic receptor P2Y_12_ was involved in the HMGB1-mediated potentiation of ADP-dependent platelet activation, we pharmacologically inhibited P2Y_12_ and TLR4, the canonical platelet receptor for HMGB1 (12, 14–17), on human platelets and measured HMBGB1- and ADP-mediated platelet activation. TLR4 inhibition had no effect on ADP-mediated platelet activation, but significantly attenuated platelet activation mediated by HMGB1 alone and in combination with ADP (Figure 2, A and B). The RAGE receptor has also been implicated in HMGB1-mediated platelet activation (14, 15), but pharmacologic inhibition of RAGE had no effect on either HMGB1- or ADP-mediated activation in our system (Supplemental Figure 2, A and B). Inhibition of P2Y_12_ with AR-C 66096 (1 μM) significantly attenuated platelet activation by ADP alone and in combination with HMGB1. However, P2Y_12_ inhibition also significantly decreased HMGB1-mediated platelet activation in the absence of ADP (Figure 2, C and D). Moreover, simultaneous inhibition of both P2Y_12_ and TLR4 caused a further reduction in HMGB1- and HMGB1 plus ADP–mediated platelet activation (Figure 2E). Importantly, none of the inhibitors significantly attenuated basal or thrombin-mediated platelet activation, confirming the lack of nonspecific effects of the antagonists. The specificity of the anti-TLR4 antibody for platelet surface TLR4 was further confirmed by its lack of effect on HMGB1-dependent platelet activation in the presence of an isotype control antibody (1 μg/mL) (Supplemental Figure 2C).

To determine whether the P2Y_12_ receptor plays a significant role in HMGB1-mediated platelet activation in vivo, we tested pharmacologic P2Y_12_ antagonism in a murine model. Mice were treated with recombinant HMGB1 (1 μg/g) after pretreatment with either vehicle or the P2Y_12_ antagonist AR-C 66096 (3 μg/g), and platelet activation was measured. Incubation with HMGB1 for 20 minutes induced significant platelet activation, and this was significantly attenuated by 15-minute pretreatment with AR-C 66096 (Figure 2F). These data demonstrate that P2Y_12_ signaling significantly contributes to HMGB1-dependent platelet activation in vitro and in vivo.

### HMGB1 increases P2Y_12_ localization at the platelet surface.

**Figure 3:**
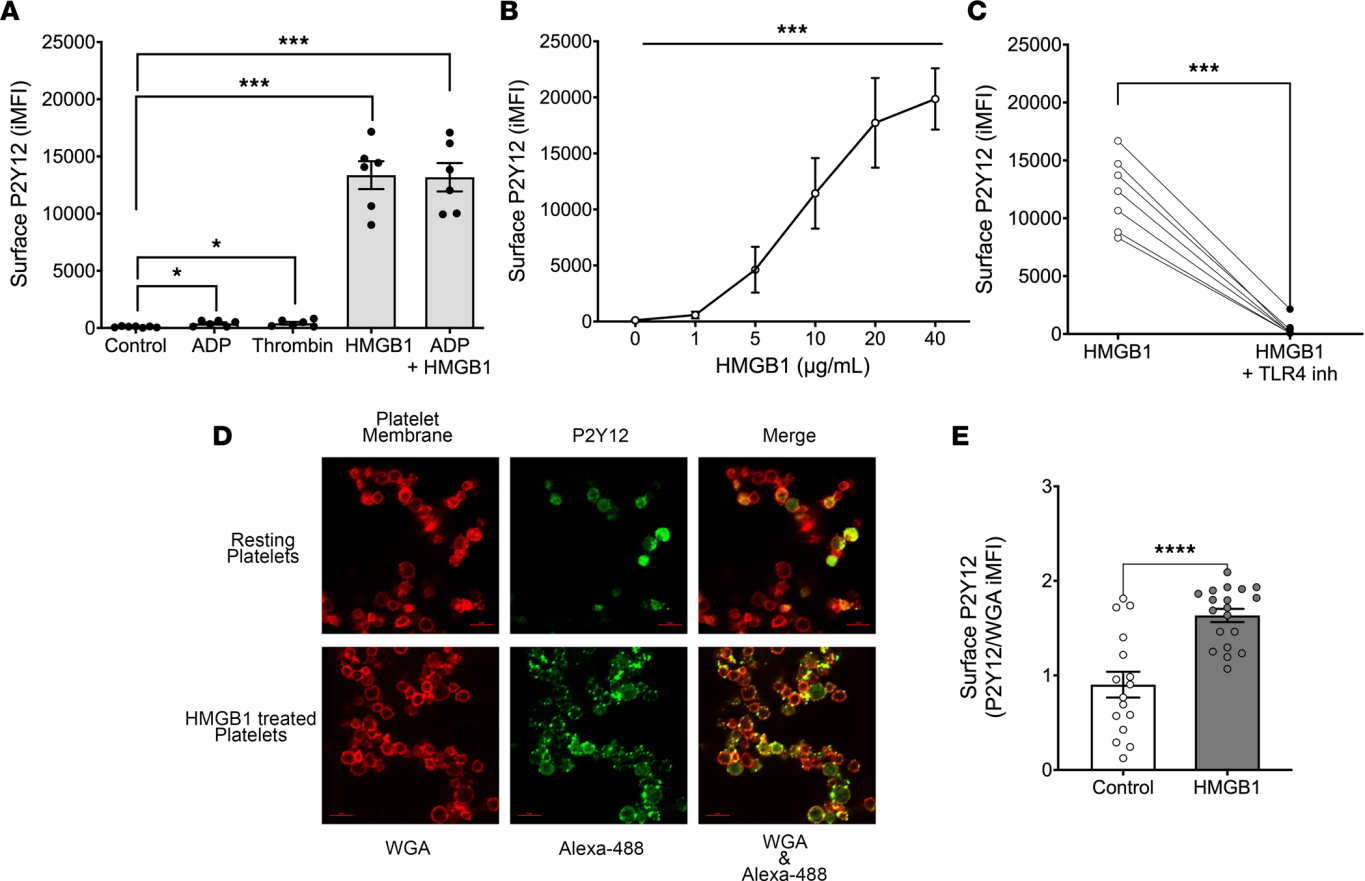
HMGB1 increases P2Y_12_ localization on the platelet surface. (**A**) P2Y_12_ on the surface of human platelets was assessed in response to no treatment (control), ADP (5 μM), thrombin (0.1 U/mL), HMGB1 (10 μg/mL), or HMGB1 plus ADP (5 μM). Data are mean ± SEM (*n* = 6) and comparisons were analyzed by paired, 2-tailed *t* test. (**B**) P2Y_12_ surface expression in response to HMGB1 (1-way ANOVA with Tukey’s post hoc test, *n* ≥ 4). Data are mean ± SEM. (**C**) Platelet surface P2Y_12_ expression with HMGB1 treatment (10 μg/mL) in the presence and absence of anti-TLR4 neutralizing antibody (1 μg/mL). *n* = 8, data are mean ± SEM and were analyzed by paired, 2-tailed *t* test. (**D**) Surface P2Y_12_ was measured on untreated (resting) (*n* = 16) or HMGB1-treated platelets (*n* = 22) by confocal microscopy. Red staining is the platelet membrane (wheat germ agglutinin, WGA). Green staining is Alexa Fluor 488 bound to anti-P2Y_12_ antibody on the cell surface (Alexa-488). Original magnification, ×63. (**E**) Corresponding quantification is depicted as platelet surface P2Y_12_ fluorescence intensity normalized to platelet membrane red WGA fluorescence intensity. Data are mean ± SEM and were analyzed by unpaired, 2-tailed *t* test. *P ≤ 0.05; ***P ≤ 0.001; ****P ≤ 0.0001.

To further investigate the role of P2Y_12_ in HMGB1-mediated platelet activation, we examined P2Y_12_ receptor levels on the membrane of human platelets. Measurement of platelet surface P2Y_12_ levels before and after (20-minute) treatment with ADP alone, HMGB1 alone, or HMGB1 plus ADP showed that ADP treatment increased surface P2Y_12_ levels. However, stimulation with HMGB1 alone or in combination with ADP resulted in a significantly greater increase in platelet surface P2Y_12_ levels (Figure 3A and Supplemental Figure 4, A and B). Consistent with these data, HMGB1 alone induced a concentration-dependent increase in platelet P2Y_12_ surface levels (Figure 3B) and this effect was attenuated by TLR4 inhibition (Figure 3C). These findings were corroborated by confocal microscopy showing increased membrane localization of P2Y_12_ after HMGB1 treatment (Figure 3, D and E). Notably, consistent with the fully oxidized and fully reduced forms of HMGB1 being weaker agonists of platelet activation than the partially oxidized protein, the fully reduced and fully oxidized HMGB1 proteins did not mediate significant translocation of P2Y_12_ to the platelet surface (Supplemental Figure 4C).

### HMGB1 induces P2Y_12_ transport to the platelet surface.

**Figure 4:**
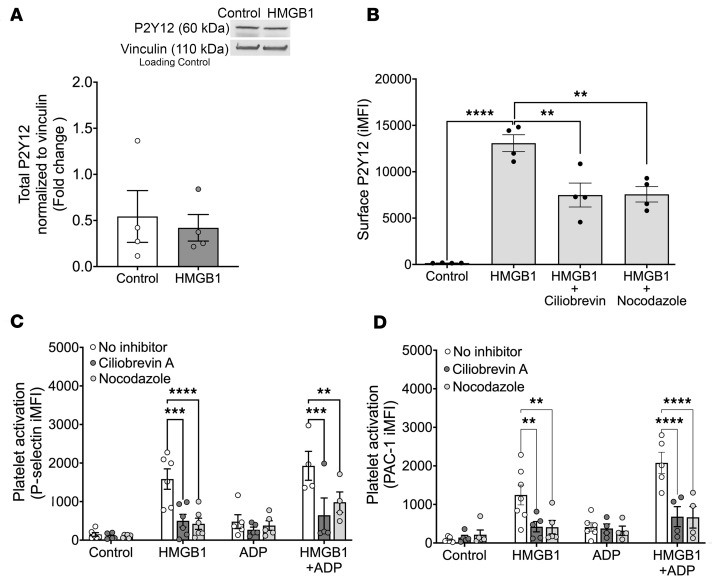
HMGB1 increases trafficking of P2Y_12_ to the platelet surface. (**A**) Total P2Y_12_ levels (surface and intracellular) in human platelets in the presence and absence of HMGB1 treatment (10 μg/mL) quantified by Western blotting. P2Y_12_ levels are displayed relative to the loading control, vinculin (*n* = 4, comparison by unpaired, 2-tailed *t* test was nonsignificant). (**B**) Platelet surface P2Y_12_ (*n* = 4) in response to no treatment (control), HMGB1 alone, or pretreatment with ciliobrevin A (10 μM), or nocodazole (2 μM) prior to HMGB1 treatment (10 μg/mL). Comparisons in **B** were analyzed by 1-way ANOVA with Tukey’s post hoc test for column-to-column comparisons. (**C** and **D**) Platelet activation measured by (**C**) surface P-selectin expression and (**D**) activated integrin αIIbβ3 (PAC-1) in response to ADP, HMGB1, or HMGB1 plus ADP after pretreatment with either ciliobrevin A or nocodazole (*n* ≥ 4). Comparisons were analyzed by 2-way ANOVA with Tukey’s multiple-comparison correction for **C** and **D**. All data are mean ± SEM. **P ≤ 0.01; ***P ≤ 0.001; ****P ≤ 0.0001.

To determine the mechanism of increased membrane P2Y_12_ in response to HMGB1, we first measured the total platelet P2Y_12_ (reflecting surface and endogenous stores) in lysed platelets, which demonstrated that total P2Y_12_ levels were unchanged by HMGB1 treatment (Figure 4A). Given that HMGB1 does not alter total P2Y_12_ protein levels, we tested whether HMGB1 increases transport of P2Y_12_ to the platelet surface. Ciliobrevin A, an inhibitor of cytoplasmic dynein, was used to disrupt dynein-mediated translocation to the surface. Pretreatment with ciliobrevin A significantly attenuated HMGB1-dependent localization of P2Y_12_ at the platelet surface (Figure 4B). Similarly, treatment with nocodazole, an inhibitor of β-tubulin–associated microtubule polymerization that is required for dynein-mediated transport, also decreased HMGB1-dependent surface P2Y_12_ levels (Figure 4B). The inhibition of cytoplasmic dynein or microtubule polymerization had no effect on ADP-mediated platelet activation (Figure 4, C and D). However, inhibition of P2Y_12_ transport significantly attenuated platelet activation stimulated by HMGB1 alone, and attenuated the HMGB1-mediated sensitization of platelets to ADP (Figure 4, C and D). These data demonstrate that HMGB1 promotes dynein-dependent transport of P2Y_12_ to the platelet surface, and that dynein-dependent transport contributes to HMGB1-induced platelet activation and underlies HMGB1-mediated sensitization of platelets to ADP.

### HMGB1-mediated platelet activation is in part due to platelet ADP release.

**Figure 5:**
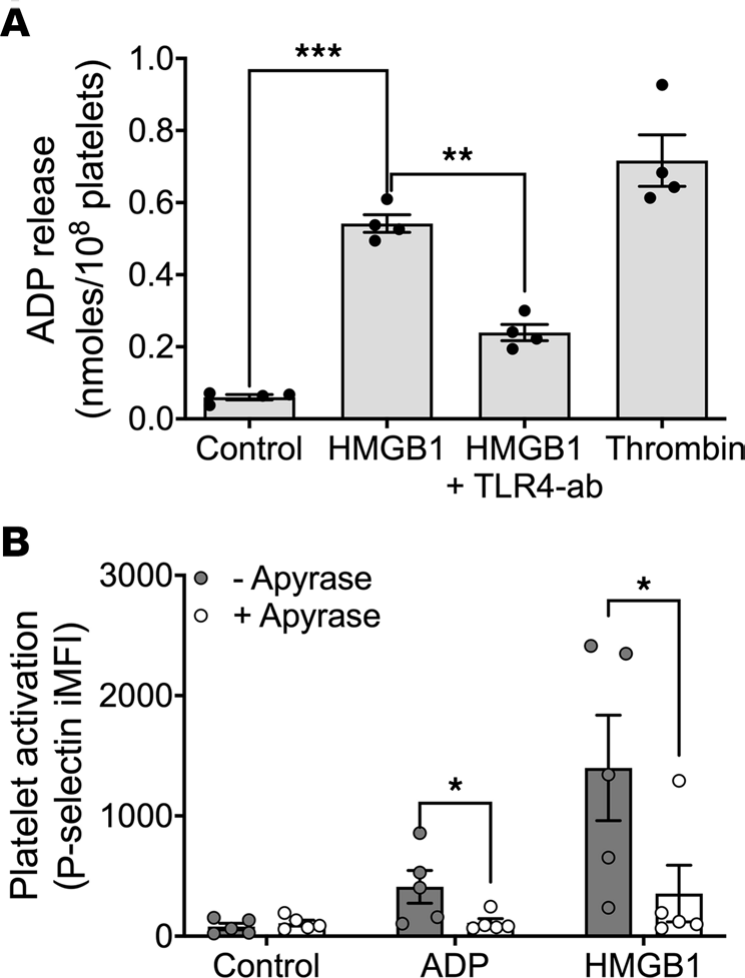
Endogenous ADP release contributes to HMGB1-dependent platelet activation. (**A**) ADP released by human platelets in response to treatment with HMGB1 (10 μg/mL) and in the presence and absence of pretreatment with TLR4-neutralizing antibody (1 μg/mL), or with thrombin control (0.1 U/mL). *n* = 4, data are mean ± SEM. Comparisons were analyzed by paired, 2-tailed *t* test. (**B**) Platelet activation stimulated by ADP (5 μM) or HMGB1 (10 μg/mL), alone or in the presence of apyrase (0.02 U/μL). *n* = 5, data are mean ± SEM. Comparisons were analyzed by 2-way ANOVA with Tukey’s correction. *P ≤ 0.05; **P ≤ 0.01; ***P ≤ 0.001.

To determine whether HMGB1 stimulates the release of platelet ADP, we measured the concentration of ADP in the supernatant of human platelets treated with HMGB1. HMBG1-treated platelets showed a significant elevation in ADP release compared with untreated platelets (Figure 5A), and this was attenuated by TLR4 inhibition (Figure 5A). Platelet CD63, a second marker of dense granule movement to the platelet surface, was also found to increase with HMGB1 stimulation (Supplemental Figure 5). To test whether the ADP released contributes to HMGB1-dependent platelet activation, we treated platelets with HMGB1 in the presence and absence of apyrase (0.02 U/μL) to degrade secreted ADP. Apyrase treatment significantly decreased HMGB1-dependent platelet activation (Figure 5B), suggesting that platelet-derived ADP contributes to HMGB1-mediated activation.

### HMGB1 contributes to elevated platelet activation in SCD.

**Figure 6:**
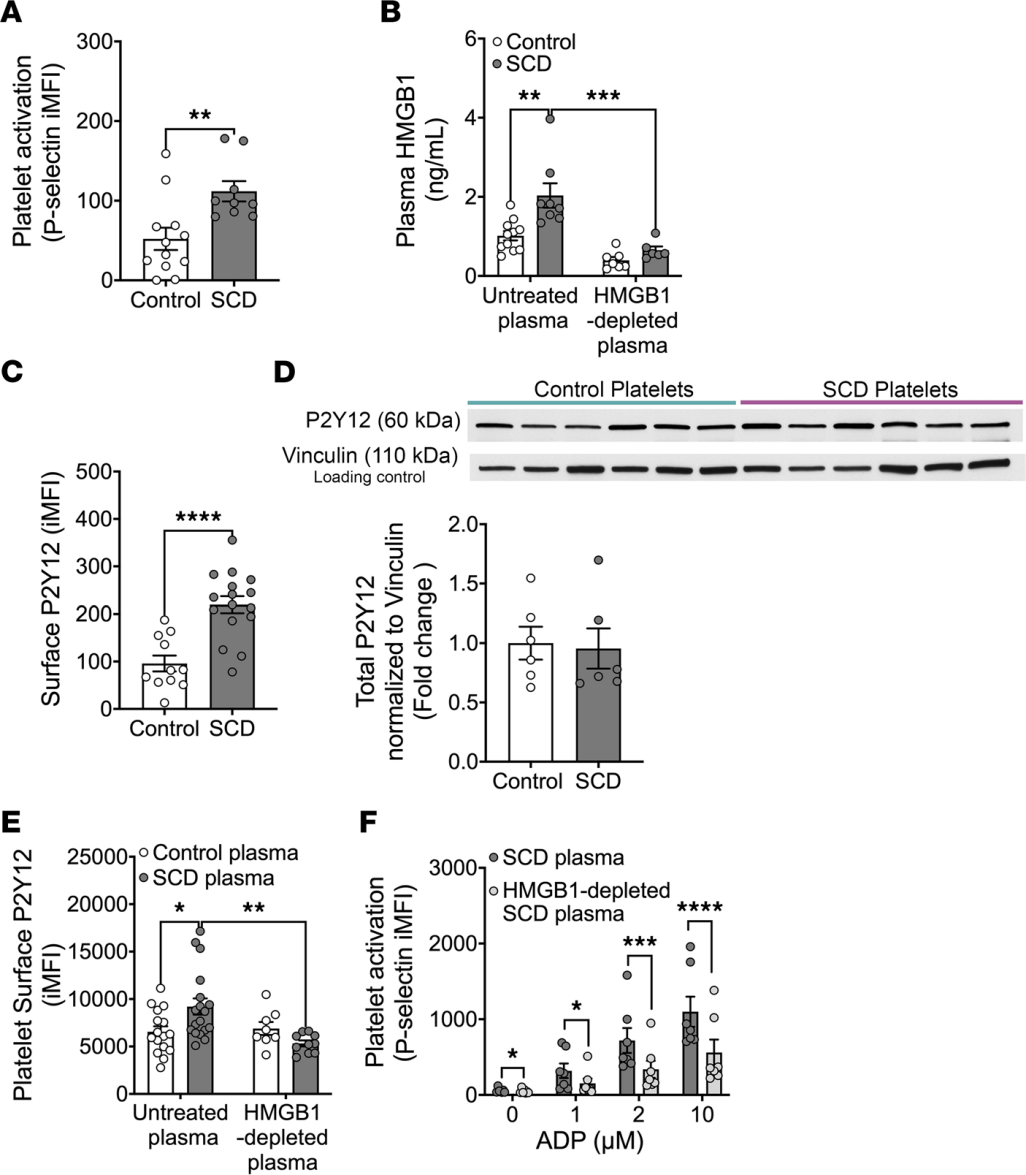
HMGB1 contributes to elevated platelet activation in sickle cell disease (SCD) through P2Y_12_ signaling. (**A**) Basal platelet activation of human SCD patients (*n* = 12) is elevated compared with control individuals (*n* = 9). Analyzed by unpaired, 2-tailed *t* test. (**B**) HMGB1 levels in plasma from SCD patients (*n* = 8) compared to controls (*n* = 11) before and after immunodepletion of HMGB1 from a subpopulation of control (*n* = 7) and SCD (*n* = 6) plasma samples (analyzed by 2-way ANOVA). (**C**) Surface P2Y_12_ on isolated human SCD platelets (*n* = 16) compared to control platelets (*n* = 11). Analyzed by unpaired, 2-tailed *t* test. (**D**) Western blot image of total P2Y_12_ levels in human platelets from patients with SCD and controls with corresponding quantification. Total platelet P2Y_12_ levels are displayed relative to vinculin loading control. *n* = 6/group. Analyzed by unpaired, 2-tailed *t* test. (**E**) Surface P2Y_12_ levels on platelets from healthy control individuals incubated with plasma from SCD patients (*n* = 18) or nonautologous control plasma (*n* = 16), or with corresponding HMGB1-depleted SCD plasma (*n* = 10) or HMGB1-depleted control plasma (*n* = 8). Analyzed by 2-way ANOVA. (**F**) ADP-induced activation of human control platelets incubated in SCD plasma with or without HMGB1 immunodepletion. *n* = 7 (2-way ANOVA). All data are mean ± SEM. *P ≤ 0.05; **P ≤ 0.01; ***P ≤ 0.001; ****P ≤ 0.0001.

We sought to determine whether HMGB1-mediated purinergic signaling contributes to elevated platelet activation in SCD. Consistent with prior studies, basal platelet activation (Figure 6A) and plasma levels of HMGB1 (Figure 6B) were higher in blood from individuals with SCD than those in blood from healthy controls. This was concomitant with greater levels of membrane P2Y_12_ in platelets from SCD patients (Figure 6C), with no difference in total platelet P2Y_12_ between SCD and control individuals (Figure 6D). To determine whether HMGB1 in SCD plasma induces platelet activation and platelet membrane P2Y_12_ localization, we treated healthy human platelets with plasma from healthy individuals or from SCD patients. Healthy human platelets treated with SCD plasma showed elevated surface P2Y_12_ (Figure 6E) and platelet activation (Figure 6F) compared with those treated with plasma from controls. Immunodepletion of HMGB1 from the SCD plasma (Figure 6B) significantly attenuated surface P2Y_12_ levels induced by the SCD plasma (Figure 6E). To determine whether the increase in P2Y_12_ induced by plasma HMGB1 sensitizes platelets to ADP-mediated activation, healthy platelets were pretreated with complete SCD plasma or the same SCD plasma depleted of HMGB1, and then treated with ADP. ADP mediated platelet activation in a concentration-dependent manner in platelets pretreated with SCD plasma, and this activation was significantly attenuated in platelets pretreated with SCD plasma depleted of HMGB1 (Figure 6F). These data demonstrate that elevated plasma HMGB1 levels in SCD increase platelet surface P2Y_12_ levels, which sensitize platelets to ADP.

## Discussion

We show for the first time to our knowledge that HMGB1 and ADP together enhance platelet activation. HMGB1-dependent TLR4 signaling releases endogenous ADP from the platelet, which potentiates platelet activation mediated by HMGB1. Concomitantly, HMGB1 increases transport of P2Y_12_ receptors to the platelet surface, which “primes” the platelet for an enhanced activation response to ADP. Furthermore, we demonstrate that this pathway is functional in individuals with SCD. These findings illustrate what we believe is a novel mechanism by which P2Y_12_ receptors are involved in inflammatory signaling and a new line of synergy between inflammatory and thrombotic pathways.

Our study demonstrates that HMGB1-associated platelet activation is partially dependent on TLR4-mediated ADP release and P2Y_12_ signaling. These data put HMGB1 in the ranks of other platelet agonists such as thrombin and collagen, which stimulate release of platelet-derived ADP to fuel feed-forward platelet activation and aggregation through P2Y_12_ (4, 51). Although ADP is considered a relatively weak physiologic agonist, it is essential for recruiting and cross-linking additional platelets and stabilizing the platelet plug (4). ADP is released from human platelets at approximately 1–1.5 nmol per 10^8^ cells (8, 52), and accumulates at micromolar levels in suspension (53–55). Our study simulated this localized ADP spike by hyperconcentrating platelets in vitro, to confirm that HMGB1 mediates both dense granule secretion and α-granule secretion. We chose to focus on the release and effect of ADP due to its interactions with P2Y_12_. However, we show that CD63 is also released, suggesting that additional dense granule molecules may be released with HMGB1 stimulation. Further study is required to determine whether other dense granule molecules released also potentiate HMGB1-mediated activation. Similarly to ADP, HMGB1 is measured at ng/mL levels in human plasma but accumulates at sites of platelet activity, and may be much higher than measured systemic levels at sites of in vivo platelet plug formation and thrombosis. This may explain why our studies required HMGB1 doses in the μg/mL range for ex vivo platelet activation (8).

Our data demonstrate that HMGB1 primes platelets for ADP-induced activation; this is consistent with a prior study in which HMGB1 caused increased sensitivity to ADP-stimulated platelet aggregation (12). We extend these studies to demonstrate that HMGB1-stimulated P2Y_12_ receptor transport mechanistically underlies this priming effect, and this transport is partially dependent on TLR4 signaling and dynein activity. Human platelets express P2Y_12_ both on their surface and in an inducible pool within the platelet that can be rapidly mobilized (56). It is estimated that about half the quantity of platelet P2Y_12_ is found on the platelet surface membrane and in the open canalicular system at all times (57), but the mechanisms that regulate platelet P2Y_12_ surface expression are not fully elucidated (58). Baurand et al. observed a 2-fold increase in membrane P2Y_12_ with ADP treatment by confocal microscopy (57), while Haberstock et al. observed a 1.3-fold increase in surface P2Y_12_ with thrombin treatment utilizing radioligand-binding studies (59). The degree of P2Y_12_ mobilization to the platelet surface was impressive in our flow cytometry studies and contrasts these other works, while our confocal data showing a 1.8-fold increase in P2Y_12_ with HMGB1 treatment is more consistent with these studies.

Our study confirms prior work demonstrating that HMGB1 induces platelet activation in a TLR4-dependent manner (15–17). Though HMGB1 has at least 11 different receptors through which it can signal (60), the 2 most prominent receptor targets for HMGB1 found on platelets are RAGE and TLR4, both of which are involved in pathways that contribute to thrombosis. HMGB1-TLR4 activity is critical for direct platelet thrombotic function (12, 14, 17, 61), and RAGE promotes thrombosis by fixing HMGB1 to the platelet surface of platelets and facilitating interactions with monocytes, NETs, and accumulation of other platelets (12, 14, 61). While RAGE plays an important role in vivo where conditions of shear flow and recruitment of surrounding cells feed a growing thrombus, HMGB1-TLR4 activity appears to drive aberrant platelet activation. For example, the prothrombotic effects of HMGB1 are nullified in TLR4-deficient mice (17), and inhibition of signaling molecules within the myeloid differentiation factor 88–dependent (MyD88-dependent) pathway downstream of TLR4 in SCD platelets inhibits platelet inflammasome activity that is involved in platelet activation and thrombus formation (13, 17). These studies are consistent with our findings that blocking TLR4 signaling instead of RAGE decreased platelet activation mediated by HMGB1. Notably, TLR4 inhibition with a TLR4-neutralizing antibody did not completely extinguish HMGB1-mediated platelet activation or HMGB1-mediated ADP release, thus highlighting an important limitation of our study. Animal models utilizing genetic modification of TLR4 are necessary to investigate what other HMGB1 platelet receptors are involved in direct platelet activation and ADP secretion.

Importantly, HMGB1 can adopt 3 discrete redox forms that engage distinct receptors. The reduced form (in which all 3 reactive thiols are reduced) mediates early inflammation and chemotaxis through stimulation of RAGE (12, 14, 61). HMGB1 can be partially oxidized, in which Cys23 and Cys45 form a disulfide bridge and the third thiol remains reduced, enabling HMGB1 to interact with TLR4 to drive peak inflammation, directly activate platelets, and propagate thrombosis (9, 15–17). Fully oxidized HMGB is inactive (62). Consistent with these prior studies, we found that the fully reduced and fully oxidized forms of HMGB1 do not stimulate platelet activation to the same degree as the partially oxidized form. Furthermore, we show that the partially oxidized form increased platelet surface P2Y_12_ to a higher degree than the other 2 forms. In our studies, platelet activation and surface P2Y_12_ induction by the partially oxidized form of the protein were mediated by TLR4 signaling. These results underscore the distinct regulatory roles of TLR4 and RAGE in mediating platelet prothrombotic responses to the distinct redox forms of HMGB1.

Our findings raise questions regarding differential TLR4 signaling in platelets. While prior studies have shown that lipopolysaccharide (LPS), a strong TLR4 agonist, may or may not directly activate platelets (63), LPS does prime human platelets to aggregate in response to ADP (64), collagen, and thrombin (63), and induces platelet dense- and α-granule release (63). Nevertheless, P2Y_12_ inhibition has shown mixed results in LPS-induced platelet activation and coagulation (65–68). Our data showing an approximately 50% decrease in HMGB1-induced platelet activation in the presence of P2Y_12_ inhibition may serve as a new foundation for studies investigating differences in LPS- and HMGB1-mediated platelet signaling. While it has been demonstrated that LPS increases expression of P2Y_12_ on macrophages (54), this type of TLR4-dependent regulation of the purinergic receptor has not been described in the platelet or with any other TLR4 ligand to the best of our knowledge. Further study is required to delineate the molecular link between TLR4 activation and the regulation of dyneins and microtubule-mediated trafficking. When surface P2Y_12_ engages ADP and reinternalizes from the platelet surface, it can either undergo degradation in the lysosome or recycle to the surface via Rab4 or Rab11, which are both dependent on cytoplasmic dynein activity along microtubules (69–71). Notably, the diversity of cytoplasmic dynein and microtubule cargos is impressive, and engagement of the microtubule system is not specific to P2Y_12_ transport (72). Rather, there is evidence that α- and dense-granule trafficking also involves the platelet microtubular system (73, 74). One limitation of our study is that the use of dynein/microtubule small molecule inhibitors, while essential for studying intracellular transport in live platelets, can have nonspecific effects on vesicle-mediated trafficking through disruption of general dynein-microtubule interactions within cells, impacting the movement of various vesicles and cargo, rather than selectively targeting a single compartment or cargo type. Of note, phosphorylation and translocation of these motor proteins within the platelet has been shown to occur after activation (75), suggesting that perhaps TLR4-dependent phosphorylation may play a role in P2Y_12_ translocation.

These data provide a potential mechanistic link for the growing number of studies that implicate P2Y_12_ in inflammatory signaling (76, 77). The platelet is an established sentinel of inflammatory signaling that not only synthesizes, stores, and releases chemokines/cytokines, but also interacts with and activates leukocytes to propagate inflammatory signaling (78). Notably, genetic or pharmacologic inhibition of P2Y_12_ decreases levels of circulating inflammatory mediators such as TNF-α, IL-10, IL-6, and MIP-1β (77) and attenuates the release of soluble CD40 ligand (19). Furthermore, P2Y_12_ inhibition attenuates platelet-neutrophil aggregates and NET formation in multiple models of LPS-induced inflammation (79, 80). Consistent with this, P2Y_12_ inhibition has shown beneficial effects in clinical studies of patients with inflammatory syndromes, including pneumonia and sepsis (76, 81). Mechanisms underlying the P2Y_12_-mediated attenuation of inflammation remain elusive and have been predominantly investigated in LPS-induced inflammatory models (79, 80). Furthermore, our data showing that pharmacological P2Y_12_ antagonism attenuates platelet activation in a murine model demonstrate that this pathway is relevant in vivo. Given that LPS can induce the release of HMGB1 to potentiate inflammatory signaling (82), the data presented here provide a rationale to delineate the crosstalk between different TLR4 agonists and ADP-dependent signaling.

Our data support the notion that P2Y_12_ potentially plays a significant role in the propagation of sterile inflammation. Pharmacologic inhibitors of P2Y_12_ are widely used therapeutically for a number of pathologies ranging from cardiovascular diseases to stroke, and rates of resistance to therapeutic P2Y_12_ inhibition have been reported as high as 30% in patients treated with clopidogrel (83). It is interesting to consider whether HMGB1-induced P2Y_12_ surface localization potentially underlies P2Y_12_ antagonist resistance, particularly in patients with inflammatory disease. Consistent with this idea, Haberstock-Debic et al. (59) found that platelet stimulation with strong agonists such as thrombin significantly increases surface P2Y_12_ expression; this increased expression was associated with clopidogrel resistance and was reversed by elinogrel, a P2Y_12_ inhibitor with longer half-life that antagonized the higher levels of receptors expressed. In pathologies with inflammatory components, acute HMGB1 release from platelets or other cells could potentially confer resistance to purinergic antagonists.

The idea that HMGB1 plays a role in P2Y_12_ antagonist resistance is highly relevant in SCD patients who have elevated levels of circulating HMGB1 and ADP. There is abundant evidence of elevated platelet activation and degranulation in SCD in vivo (84, 85); preclinical and early-phase studies have demonstrated that P2Y_12_ antagonists decrease platelet activation and inflammatory signaling in murine models and human in vitro models of SCD (47–49). Despite this recognition, human trials studying the clinical utility of platelet inhibitors, including P2Y_12_ antagonists, have been inconclusive (49, 86–89). Primary endpoints in these human studies have focused mainly on reducing vaso-occlusive pain, transfusion needs, and episodes of acute chest syndrome, rather than on thrombotic outcomes or measurement of inflammatory signaling (44). Ticlopidine was effective in reducing acute pain episodes in SCD patients after 6 months of treatment (45), while prasugrel inhibited platelet activation in adults with SCD, but did not reduce pain episodes (46). In children with SCD, prasugrel did not affect vaso-occlusive rates overall, but showed potential benefits in 12- to 17-year-old children not taking hydroxyurea (90). Large-scale studies on the efficacy of P2Y_12_ antagonists to prevent thrombotic and inflammatory complications in SCD are lacking (3, 91), and further studies that include assessment of circulating levels of HMGB1 may assist in reconciling different outcomes found for P2Y_12_ inhibitors within prior studies.

In summary, this study shows for the first time to our knowledge that ADP release and P2Y_12_ activation contribute to HMGB1-induced platelet activation, and that HMGB1 primes platelets for ADP signaling by increasing P2Y_12_ at the platelet surface without invoking new P2Y_12_ synthesis. These data present what we believe is a novel mechanism of crosstalk between inflammatory and thrombotic signaling and have implications for both the therapeutic targeting of HMGB1 and P2Y_12_ receptors in healthy individuals and those with SCD and other thromboinflammatory diseases.

## Methods

Additional details can be found in the Supplemental Methods.

### Sex as a biological variable.

Individuals of both sexes were recruited for human studies, and no significant difference was found in the responses of males and females. Male mice were exclusively used for murine studies, but based on human data, these data should be applicable to female mice as well.

### Reagents.

For a comprehensive list of antibodies/reagents utilized and sources, see Supplemental Table 1. Recombinant HMGB1 (R&D Systems) was used throughout the study unless otherwise noted. The recombinant protein used is partially oxidized, with a disulfide bond between Cys23 and Cys45 and a free Cys106 thiol on the HMGB1 protein (50, 92).

### Human participants.

Participants consisted of 47 healthy adults (mean age 38 years) and 18 adult patients with SCD (HbSS or HbS/β^0^ thalassemia, mean age 33 years) in steady state and on hydroxyurea. Steady state was defined as at least 4 weeks since hospitalization or blood transfusion. Those under 18 years old, pregnant, or taking antiplatelet or anticoagulant medications were excluded.

### Mouse studies.

Male C57BL/6J mice (Jackson Laboratory) aged 10–12 weeks (20–30 grams) received a tail vein injection of AR-C 66096 or vehicle control, and 60 minutes later received HMGB1 by tail vein injection. Murine blood was collected via cardiac puncture in sodium citrate with a 9:1 blood/anticoagulant ratio, and platelets were isolated to assess activation as described below.

### Isolation of platelets.

Human blood was collected in sodium citrate by venipuncture using light or no tourniquet. Platelets were isolated by differential centrifugation as previously described (93). See the Supplemental Methods.

### Measurement of platelet activation and surface P2Y_12_.

Platelets (2 × 10^7^ cells/mL) were treated with agonist (see Supplemental Methods) and fixed with 1% paraformaldehyde and incubated with fluorescent antibodies against the markers integrin αIIb (CD41a, PE-labeled), the activation-dependent conformation of integrin αIIbβ3 (PAC-1, FITC-labeled), P-selectin (CD62P, APC-labeled for human samples, FITC-labeled for mouse samples), and P2Y_12_ (FITC-labeled). Platelets were also gated using forward and side scatter. Anti-CD41a antibody was used to identify purified platelets. Platelet activation was defined as the population of cells that were (a) of an appropriate size for platelets using forward and side scatter gates, (b) positive for CD41a, and (c) positive for either P-selectin (CD62P) or activated integrin αIIbβ3 (PAC-1). Surface P2Y_12_ expression was defined as the population of cells that were (a) of an appropriate size using forward and side scatter gates, (b) positive for CD41a, and (c) positive for anti-P2Y_12_ antibody. Examples of the gating strategy can be found in Supplemental Figure 3. A minimum of 50,000 events per sample was acquired.

Platelet activation and surface P2Y_12_ were derived as both percentage and mean fluorescence intensity (MFI) values. The integrated MFI (iMFI) was calculated to represent the total platelet response to a stimulus. In flow cytometry studies of cell-mediated biomarkers, iMFI is a preferred correlate of function over either the percentage or the MFI of the positive population because it accounts for both the magnitude (%) and the quality (raw MFI) of the data to demonstrate the population of platelets expressing a particular marker (94–97). iMFI values were derived by multiplying the relative frequency (%) of cells expressing both CD41a and (a) P-selectin, (b) activated integrin αIIbβ3, or (c) P2Y_12_, by the raw MFI of that same population (94). Flow cytometry data were acquired using a Fortessa fluorescence-activated cell sorter with Diva Software (Becton Dickinson) and analyzed using Flowjo version 10.0.

### Measurement of platelet ADP release.

Isolated human platelets (3 × 10^8^ cells/mL) were treated and then centrifuged (1500*g*, 5 minutes) in the presence of prostaglandin I_2_ (1 μg/mL; Cayman Chemical, 18220). ADP levels in the supernatant were quantified by ELISA (BioVision ADP Colorimetric/Fluorometric Assay Kit, K355).

### Surface platelet P2Y_12_ imaging.

Isolated human platelets (2 × 10^7^ cells/mL) were treated and fixed with 4% paraformaldehyde and stained with primary rabbit anti-P2Y_12_ monoclonal antibody (1:100 dilution) and secondary donkey anti-rabbit IgG Alexa Fluor 488 (1:10,000 dilution). The platelet cell membrane was identified by red wheat germ agglutinin (WGA, 1:1000 dilution). Images of mounted platelets were captured by fluorescence microscopy (Nikon A1 confocal microscope). There were 30 view fields per experiment analyzed with each section size measuring 0.2 μm. NIS-Elements was used to calculate raw MFI and quantify P2Y_12_-positive surface clusters, from which iMFI was calculated. Surface P2Y_12_ colocalized to red WGA was calculated as iMFI, where iMFI = number of Alexa Fluor 488–positive clusters per platelet × raw MFI.

### HMGB1 immunodepletion.

Magnetic beads coated with anti-HMGB1 antibody (Sino Biological, MB100930-T46) were used to deplete HMGB1 from platelet-poor plasma following the manufacturer’s instructions.

### Measurement of HMGB1 concentration.

Platelet-poor plasma was isolated (see Supplemental Methods), and HMGB1 levels were quantified by ELISA (Tecan, ST51011) in accordance with the manufacturer’s instructions.

### Quantification of total P2Y_12_.

Isolated human platelets (4 × 10*^7^* cells/mL) were lysed by repeated freeze-thaw, and Western blotting was performed as previously described (98, 99).

### Statistics.

Human samples represent different biological replicates (platelets isolated from the blood of different individuals). In assays involving paired measurements with inhibitors of platelet activation or P2Y_12_ localization, statistical significance was determined by paired parametric *t* test unless otherwise indicated. Murine samples were analyzed by 1-way ANOVA with Tukey’s post hoc test. *P* values of less than 0.05 were considered significant. Analyses were performed using GraphPad Prism 9 software. Data are presented as mean ± standard error of the mean (SEM) unless otherwise specified.

### Study approval.

This study was approved by the University of Pittsburgh Institutional Review Board (IRB) and performed in accordance with the relevant regulations. Written informed consent was obtained from all participants. All murine studies were performed in accordance with approval by the University of Pittsburgh Institution Animal Care and Use Committee.

### Data availability.

The authors confirm that the data supporting the findings of this study are available within the article, its supplemental materials, the Supporting Data Values file, or from the corresponding author (SS) upon reasonable request.

## Author contributions

DND, GKA, and SS designed the study. DND and GKA performed the research. DND, GKA, CSC, MC, and SS analyzed the data. DND, SS, and CAH wrote the manuscript.

## Supplementary Material

Supplemental data

Unedited blot and gel images

Supporting data values

## Version 1 03/08/2024

Electronic publication
