# Supplementary material for: High-mobility group box 1 increases platelet surface P2Y12 and platelet activation in sickle cell disease
Source: JCI Insight. 2024 Mar 8;9(5):e174575. doi: 10.1172/jci.insight.174575 (PMC10972595; doi:10.1172/jci.insight.174575)
Supplement: Supplemental data [file jciinsight-9-174575-s256.pdf]

Supplementary Information File

High-Mobility Group Box 1 increases platelet surface $P2Y_{12}$ and platelet activation in sickle cell disease

Deirdre Nolfi-Donegan[1,2,3≠], Gowtham K Annarapu[3≠], Claudette St. Croix[4], Michael Calderon[4], Cheryl A Hillery[1,2], and Sruti Shiva[3,5,6*]

[1]*Department of Pediatrics, Division of Hematology/Oncology, Children's Hospital of Pittsburgh, Pittsburgh, PA, USA, 15621*
[2]*University of Pittsburgh School of Medicine, University of Pittsburgh, Pittsburgh, PA, USA, 15261*
[3]*Pittsburgh Heart, Lung, Blood Vascular Medicine Institute, University of Pittsburgh, Pittsburgh, PA, USA, 15261*
[4]*Center for Biologic Imaging, University of Pittsburgh, Pittsburgh, PA, USA, 15261*
[5]*Center for Metabolism and Mitochondrial Medicine, University of Pittsburgh, Pittsburgh, PA, USA 15261*
[6]*Department of Pharmacology & Chemical Biology, University of Pittsburgh, Pittsburgh, PA, USA, 15261*

[≠]These authors contributed equally

*Corresponding:
Sruti Shiva, PhD
E1220 Thomas E. Starzl Biomedical Science Tower
200 Lothrop Street, Pittsburgh, PA 15213
Phone 412-383-5854
Fax 412-648-5980
E-mail address: sss43@pitt.edu

Running head: HMGB1 enhances ADP-mediated platelet activation

Supplemental Methods

Isolation of human and murine platelets

Blood was centrifuged (500*g*, 20 minutes, 25°C) to obtain platelet-rich plasma (PRP). PRP was centrifuged (1500*g*, 10 minutes, 25°C) in the presence of PGI_2 (1 µg/mL) to separate platelets from platelet-poor plasma (PPP), which was collected and stored separately. The platelet pellet was washed in erythrocyte lysis buffer containing PGI_2, centrifuged again (1500*g*, 5 minutes, 25°C), and resuspended in platelet buffer, which was a modified Tyrodes buffer (20mmol/L HEPES, 128mmol/L NaCl, 12mmol/L sodium bicarbonate, 0.4mmol/L NaH_2PO_2, 5mmol/L glucose, $CaCl_2$ 3mmol/L, 1 mmol/L $MgCl_2$, 2.8mmol/L KCl, pH 7.4). Platelet purity was confirmed by flow cytometric measurement of CD41a expression.

Measurement of platelet activation and surface $P2Y_{12}$

Isolated (washed) human platelets were incubated (20 min; 25°C) with agonists including human recombinant HMGB1 (R&D Systems), ADP (Bio/Data Corporation), collagen (Bio/Data Corporation), thrombin enzyme (Sigma-Aldrich), or vehicle control. In assays involving inhibition of specific receptors, platelets were pre-treated with inhibitors (15-30 min) prior to agonist. Targets for inhibition were TLR4 (anti-TLR4 IgG and its IgG1 isotype control; InvivoGen); RAGE (FPS-ZM1; Calbiochem); $P2Y_{12}$ (AR-C 66096; Tocris); cytoplasmic dynein (ciliobrevin A; R&D Systems); and β−tubulin (nocodazole; Sigma-Aldrich). In assays involving plasma incubation of platelets, platelets were isolated and washed prior to plasma exposure.

Measurement of oxidation status of HMGB1 by biotin switch assay

A biotin labeling assay was used to investigate the oxidation state of the cysteine residues on recombinant HMGB1 (R&D Systems) as previously described(1). Briefly, recombinant HMGB1 was either reduced by sodium dithionite (100 mM, 15 min), oxidized by hydrogen peroxide (H_2O_2, 100 µM, 15 min), or left untreated. The exposed free thiol groups in each of these samples were labeled with EZ-Link Maleimide-PEG2-Biotin,

100μM (Thermo Scientific) by incubating for 2 hours at 40°C. Samples were loaded onto 10% SDS page gel and electrophoresis was performed as described(98). Biotin was detected by streptavidin binding (1:5000, Li-Cor) images were captured by Odyssey CLx Imager (Li-Cor). Semiquantitative analysis was performed using Image Studio Lite software. Biotin quantification was normalized to quantification of HMGB1 protein within each lane (using monoclonal anti-HMGB1 antibody 1:500 dilution, Biolegend). This approach provided insight into the relative oxidation level of recombinant HMGB1 used throughout the manuscript and allowed for semiquantitative analysis of the biotin-labeled thiol groups.

Quantification of total $P2Y_{12}$ in human platelets by Western Blot

Isolated human platelets (4×10^7 cells/*m*L) were treated with either HMGB1 (10 μg/ml) or vehicle for 20 minutes at room temperature and were lysed by repeated freeze-thaw. SDS-PAGE gel electrophoresis was performed as described(98). After transfer to nitrocellulose, membranes were blocked with 5% milk in TBST. $P2Y_{12}$ was probed with primary rabbit anti-$P2Y_{12}$ mAb (1:1000 dilution) in 2.5–5% milk and incubated at 4°C overnight; rabbit anti-vinculin mAb (1:500 dilution; ThermoFisher Scientific #700062) was probed as loading control. Membranes were washed and incubated with horseradish peroxidase conjugated secondary antibody followed by ECL reagent as reported(99). Images were captured by chemiluminescence on a Kodak X-OMAT 2000 Processor. Semiquantitative analysis was performed using Image Lite software.

Supplemental Table 1: Key Antibodies and Reagents

**Antibodies**		
**Item**	**Vendor**	**Catalog reference**
Anti-hTLR4-IgG	InvivoGen	mabg-htlr4
Isotype Ctrl IgG1 for TLR4 ab	InvivoGen	mabg1-ctrlm
FITC anti-human P2RY12 Antibody	Biolegend	392108
PE Mouse Anti-Human CD41a (GPIIb)	BD Biosciences	555467
APC Mouse Anti-Human CD62P	BD Biosciences	550888
FITC Mouse Anti-Human PAC-1 (activated integrin αIIbβ3)	BD Biosciences	340507
Alexa Fluor® 488 anti-human CD41 Antibody	Biolegend	303724
Integrin alpha 2b/CD41 Antibody (M148) [Alexa Fluor® 488]	Novus Biologicals	NB100-2614AF488
BV421 Mouse Anti-Human CD63	BD Biosciences	740080
Ultra-LEAF™ Purified anti-HMGB1 antibody	Biolegend	651413
P2Y12 Recombinant Rabbit Monoclonal Antibody (4H5L19)	Invitrogen	702516
Donkey anti-Rabbit IgG (H+L) Highly Cross-Adsorbed Secondary Antibody, Alexa Fluor™ 488	Invitrogen	A-21206
Vinculin Recombinant Rabbit Monoclonal Antibody (42H89L44)	Invitrogen	700062
PE Rat Anti-Mouse CD41	BD Biosciences	558040
FITC Rat Anti-Mouse CD62P	BD Biosciences	553744
APC anti-P2RY12 Antibody	Biolegend	848006
**Other reagents**		
**Item**	**Vendor**	**Catalog reference**
RAGE Antagonist, FPS-ZM1	Calbiochem	553030
AR-C 66096 tetrasodium salt (P2Y12 antagonist)	Tocris	3321/1
Ciliobrevin A	R&D Systems	4529
Nocodazole	Sigma	M1404
Apyrase from potatoes, ATPase ≥200 units/mg protein, lyophilized powder	Sigma Aldrich	A6410
Wheat Germ Agglutinin, Texas Red™-X Conjugate	Invitrogen	W21405
EZ-Link Maleimide-PEG2-Biotin	Thermo Fisher	21902
IRDye® 800CW Streptavidin	Li-Cor	926-32230
**Detection kits**		
**Item**	**Vendor**	**Catalog reference**
HMGB1 ELISA	Tecan	ST51011
ADP Colorimetric/Fluorometric Assay Kit	BioVision	K355
**Agonists**		
**Item**	**Vendor**	**Catalog reference**
Bio/Data™ ADP, lyophilized	Fisher Scientific	22-515-225
Bio/Data™ collagen	Fisher Scientific	101562
Thrombin from human plasma	Sigma-Aldrich	T6884
Recombinant Human HMGB1 Protein, CF	R&D Systems	1690-HMB-050

Legend for Table S1: A comprehensive list of key reagents and antibodies used within this study are listed here.

Supplemental Figures & Legends

Supplemental Figure S1

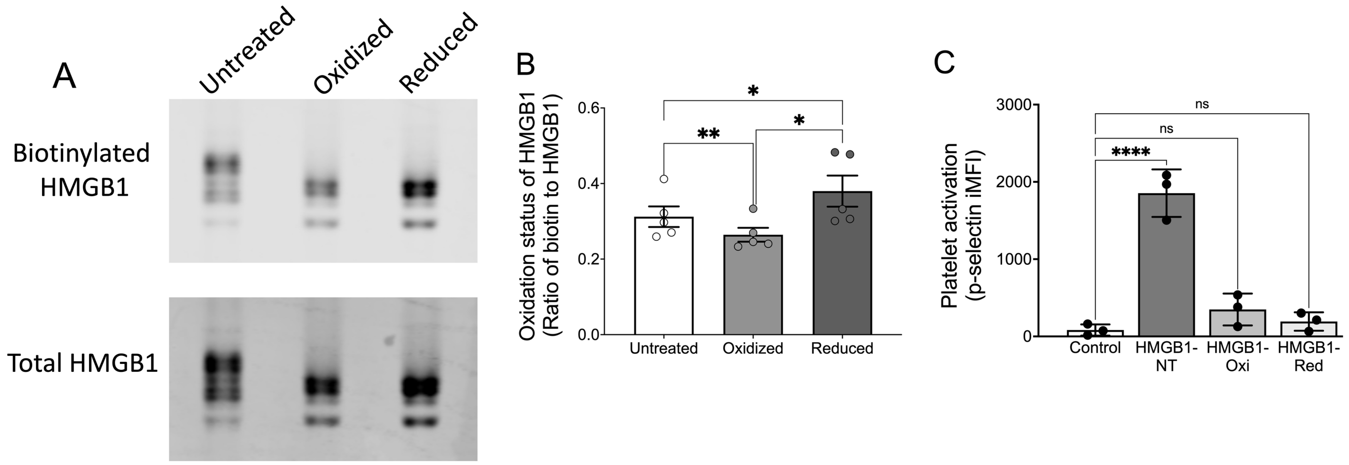

Supplemental Figure S1: Evaluating the impact of the redox status of recombinant HMGB1 on platelet activation. (A) Biotin signal normalized to HMGB1 protein for untreated recombinant HMGB1 (R&D Systems) and for recombinant HMGB1 that was reduced and oxidized, respectively, and (**B**) corresponding quantifications. Higher biotin signal denotes more free thiols (SH) as seen with the reduced form. Lower and intermediate biotin signals are expected for the oxidized and disulfide forms, respectively, where the sulfonate groups (HO3S) and disulfide S-S bond limits available binding sites. n=5 and are analyzed by one-way ANOVA. (**C**) Platelet activation with stimulation of each redox form of HMGB1 (10 μg/ml). n=3 and are analyzed by one-way ANOVA. All data are mean ± SEM * P ≤ 0.05; ** P ≤ 0.01; **** P ≤ 0.0001.

Supplemental Figure S2

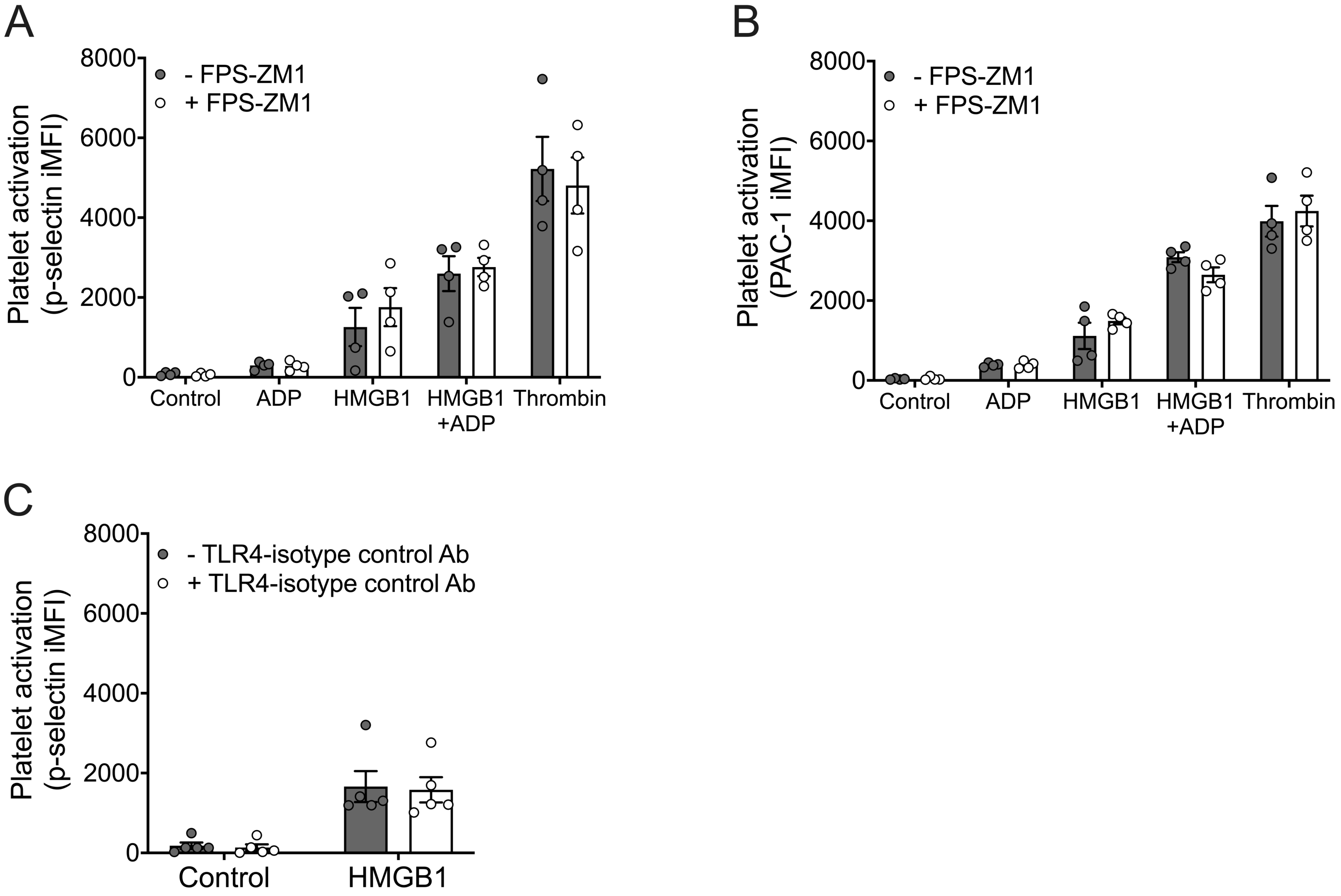

Legend for Supplemental Figure S2: RAGE inhibition and inhibition with isotype control for the TLR4 neutralizing antibody do not attenuate HMGB1-dependent activation in human platelets. (**A-B**) Platelet activation measured by p-selectin and activated integrin αIIbβ3 (PAC-1) by integrated mean fluorescent intensity (iMFI) at baseline and after stimulation with ADP alone (5 μM), HMGB1 alone (10 μg/ml), HMGB1+ADP, or thrombin alone (0.1 U/ml), and combined with pre-treatment with RAGE inhibitor FPS-ZMI (25 nM), (**C**) with or the isotype control antibody for anti-TLR4 neutralizing antibody (1 μg/ml, n=5). n=4, data are mean ± SEM and comparisons are analyzed by paired t-test. No paired comparisons are significant.

Supplemental Figure S3

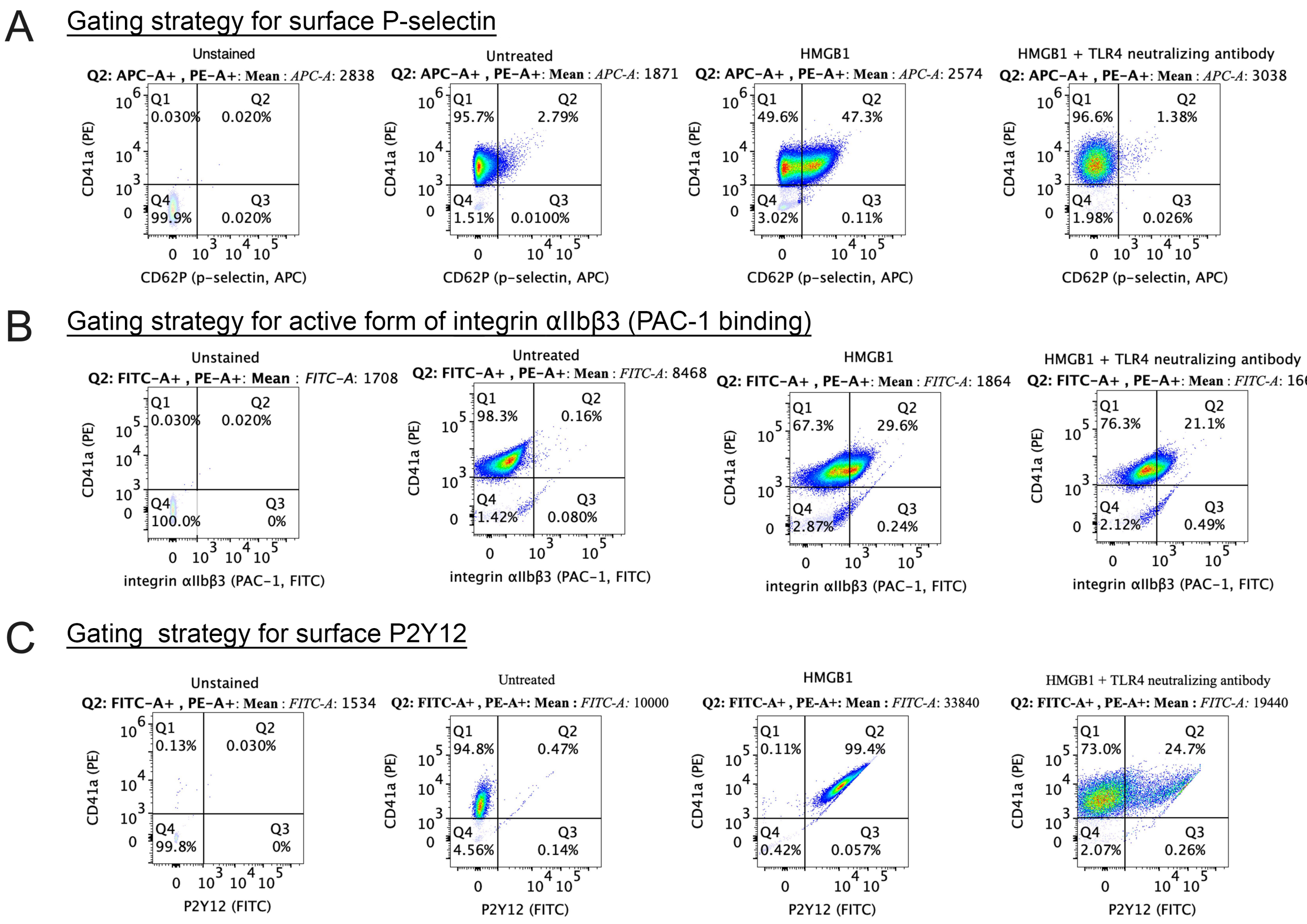

Legend for Supplemental Figure S3: Platelets were gated according to forward and side scatter and identified with fluorescent antibody markers to platelet surface proteins. The data depicted are examples of platelets unstained, untreated (baseline), HMGB1-treated, or treated with an inhibitor (TLR4 neutralizing antibody in this example), for **(A)** surface p-selectin, **(B)** activated integrin αIIbβ3 (PAC-1), or **(C)** surface $P2Y_{12}$. Each plot depicts the raw percentage and raw mean fluorescent intensity of the double-positive population (Q2) which was used to calculate integrated mean fluorescent intensity (iMFI).

Supplemental Figure S4

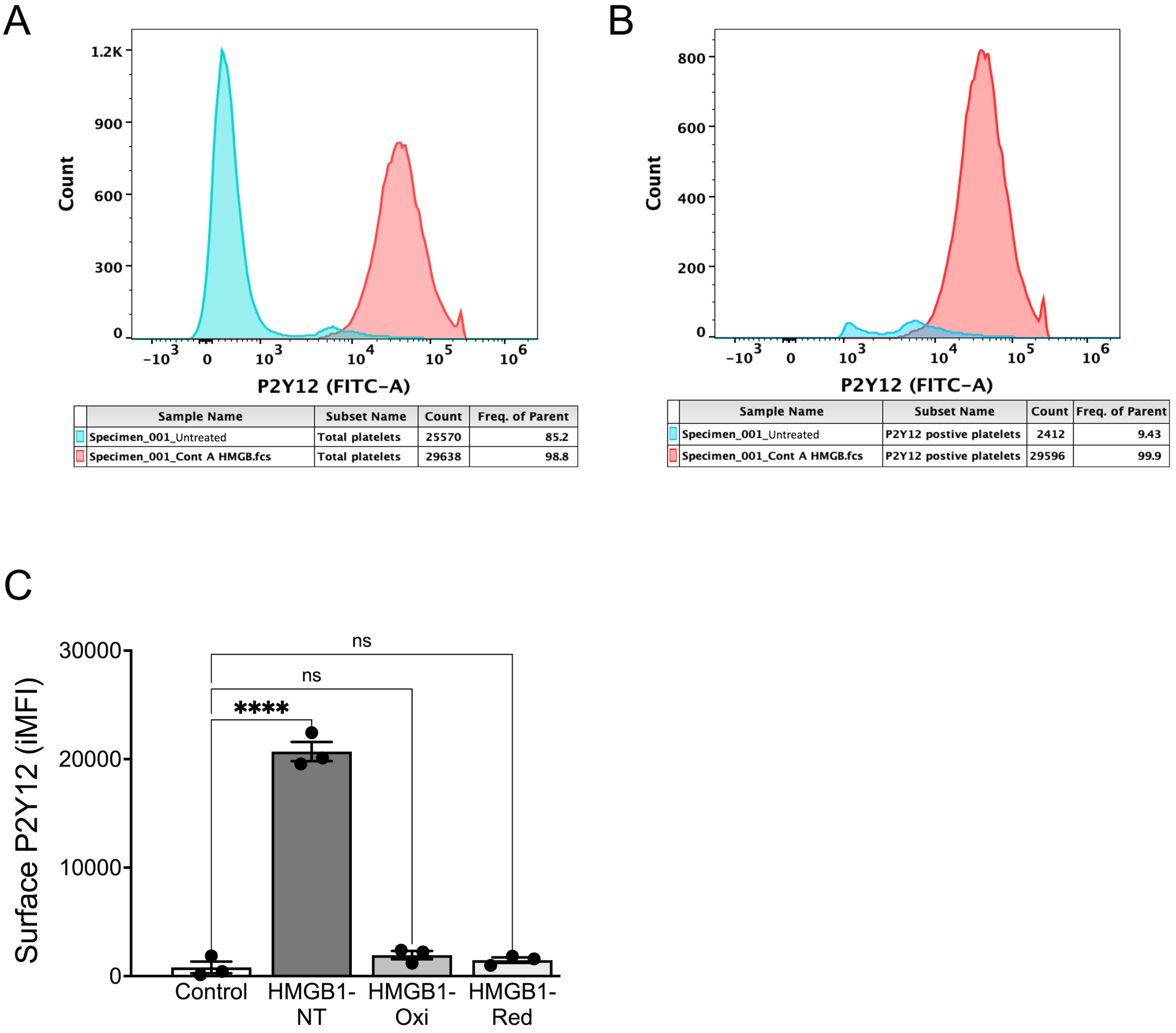

Legend for Supplemental Figure S4: Platelet surface P2Y$_{12}$ with HMGB1 treatment. (**A**) Surface P2Y$_{12}$ is heterogeneously distributed amongst two populations of resting, untreated platelets as evidenced by one large blue peak negative for P2Y$_{12}$ and a smaller blue peak that is positive, whereas there is only one positive population for recombinant HMGB1-treated platelets (red peak). (**B**) The population of platelets that is double-positive for both CD41a and P2Y$_{12}$ is demonstrated for resting platelets (blue peaks) and recombinant HMGB1-treated platelets (red peak). (**C**) Platelet surface P2Y$_{12}$ with stimulation of each redox form of HMGB1 (10 µg/ml): non-treated recombinant (NT), oxidized recombinant (Oxi), and reduced recombinant (Red). n=3 and are analyzed by 1-way ANOVA. Data are mean ± SEM. ns=not significant; * P ≤ 0.05; ** P ≤ 0.01; **** P ≤ 0.0001.

Supplemental Figure S5

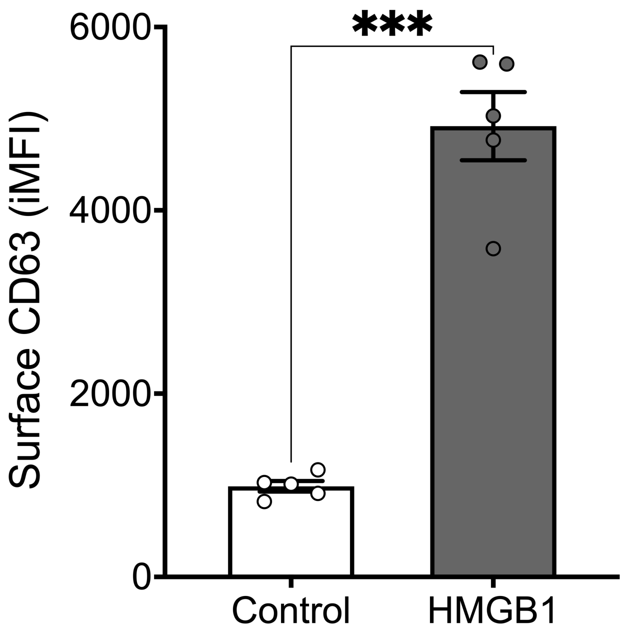

Legend for Supplemental Figure S5: HMGB1 increases the presence of dense granule marker CD63 at the platelet surface. Surface CD63 depicted as integrated mean fluorescent intensity (iMFI) at baseline and after stimulation with HMGB1 (10 μg/ml). n=5. Data are mean ± SEM and comparisons are analyzed by paired t-test. *** P ≤ 0.001.

REFERENCES

1. Forrester MT, Foster MW, Benhar M, and Stamler JS. Detection of protein S-nitrosylation with the biotin-switch technique. Free Radic Biol Med. 2009;46(2):119-26.
