# Supplementary material for: High-mobility group box 1 increases platelet surface P2Y12 and platelet activation in sickle cell disease
Source: JCI Insight. 2024 Mar 8;9(5):e174575. doi: 10.1172/jci.insight.174575 (PMC10972595; doi:10.1172/jci.insight.174575)
Supplement: Unedited blot and gel images [file jciinsight-9-174575-s257.pdf]

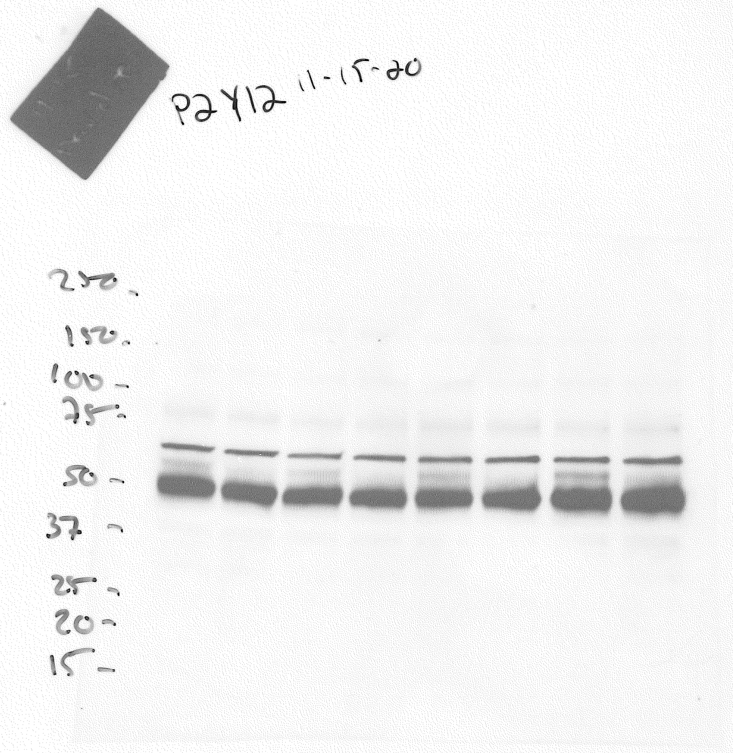


Full unedited gel for Figure 4A, depicting P2Y12 at ~ 60 kda and nonspecific band at ~50 kda. Lanes 1 and 2 of the unedited gel correspond to those treatments shown in the cropped images within the manuscript: “Control” and “HMGB1”.

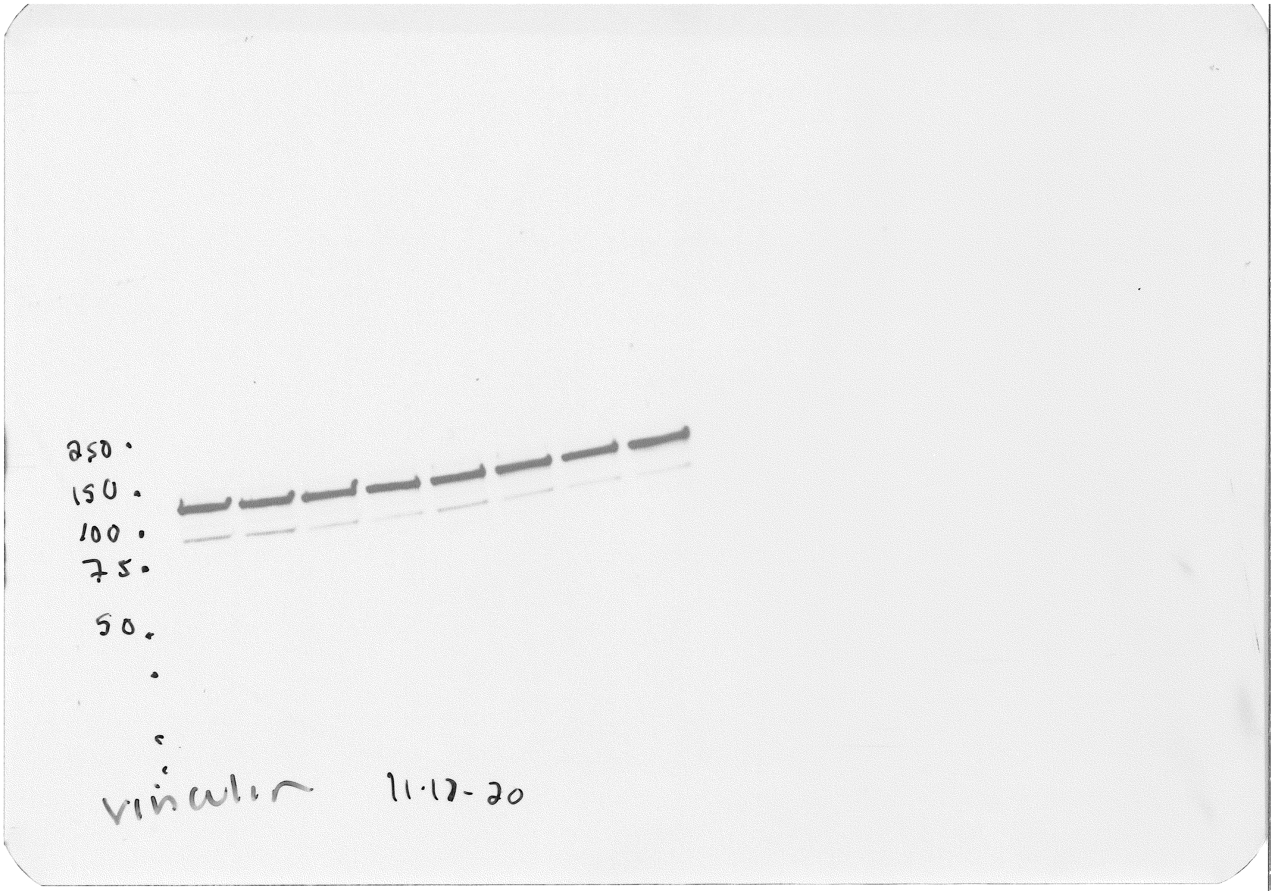


Full unedited gel for Figure 4A, depicting vinculin loading control at ~ 110 kda. Excess bands at ~100 kda on the loading control membrane represent additional antibodies not included in the final version of this manuscript. Lanes 1 and 2 of the unedited gel correspond to those treatments shown in the cropped images within the manuscript: “Control” and “HMGB1”.

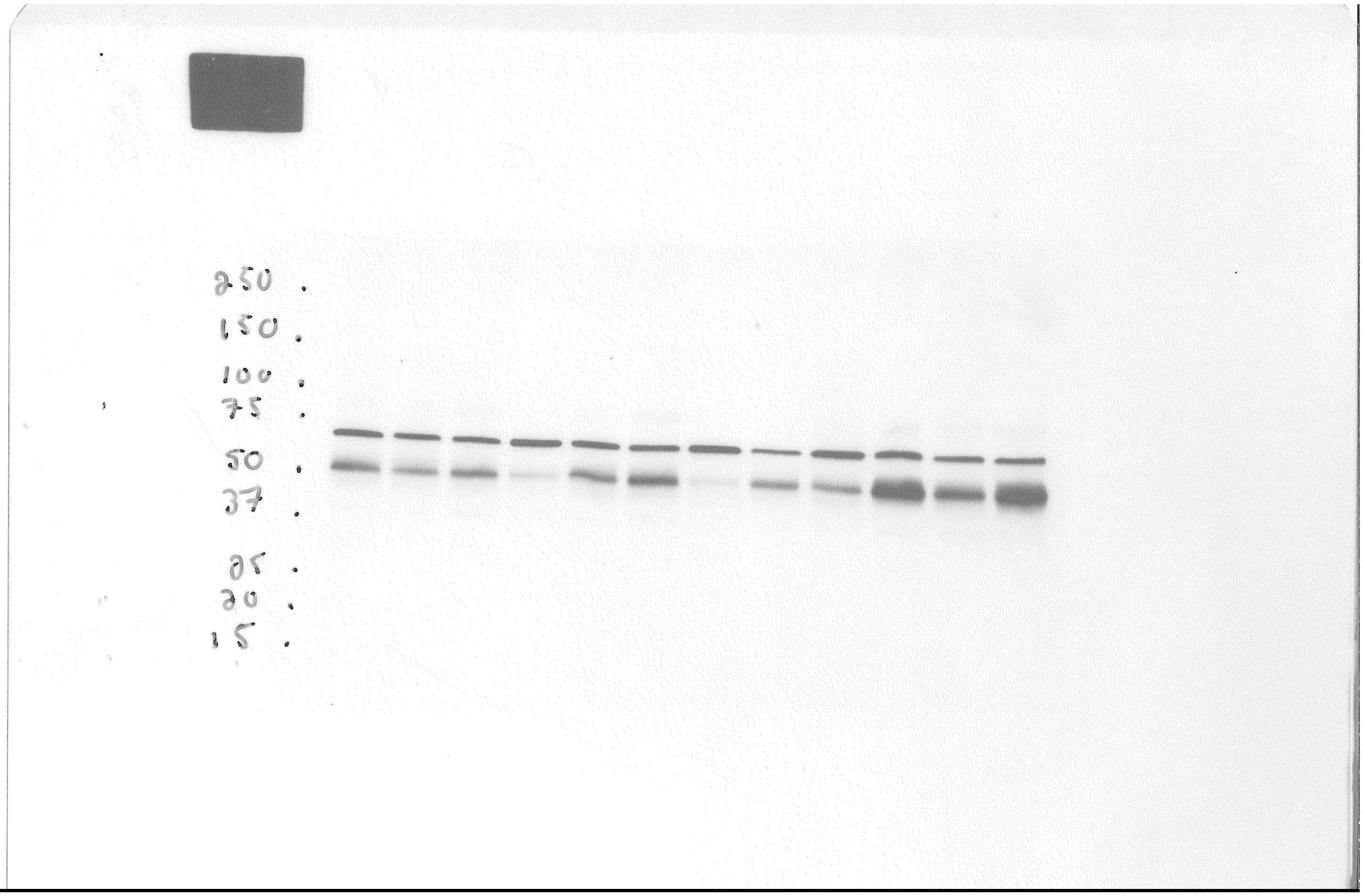


Full unedited gel for Figure 6D, depicting P2Y12 at ~ 60 kda and nonspecific band at ~50 kda. Lanes 1-6 and 7-12 of the unedited gel correspond to those treatments shown in the cropped images within the manuscript: “Control” (Lanes 1-6) and “SCD” (Lanes 7-12).

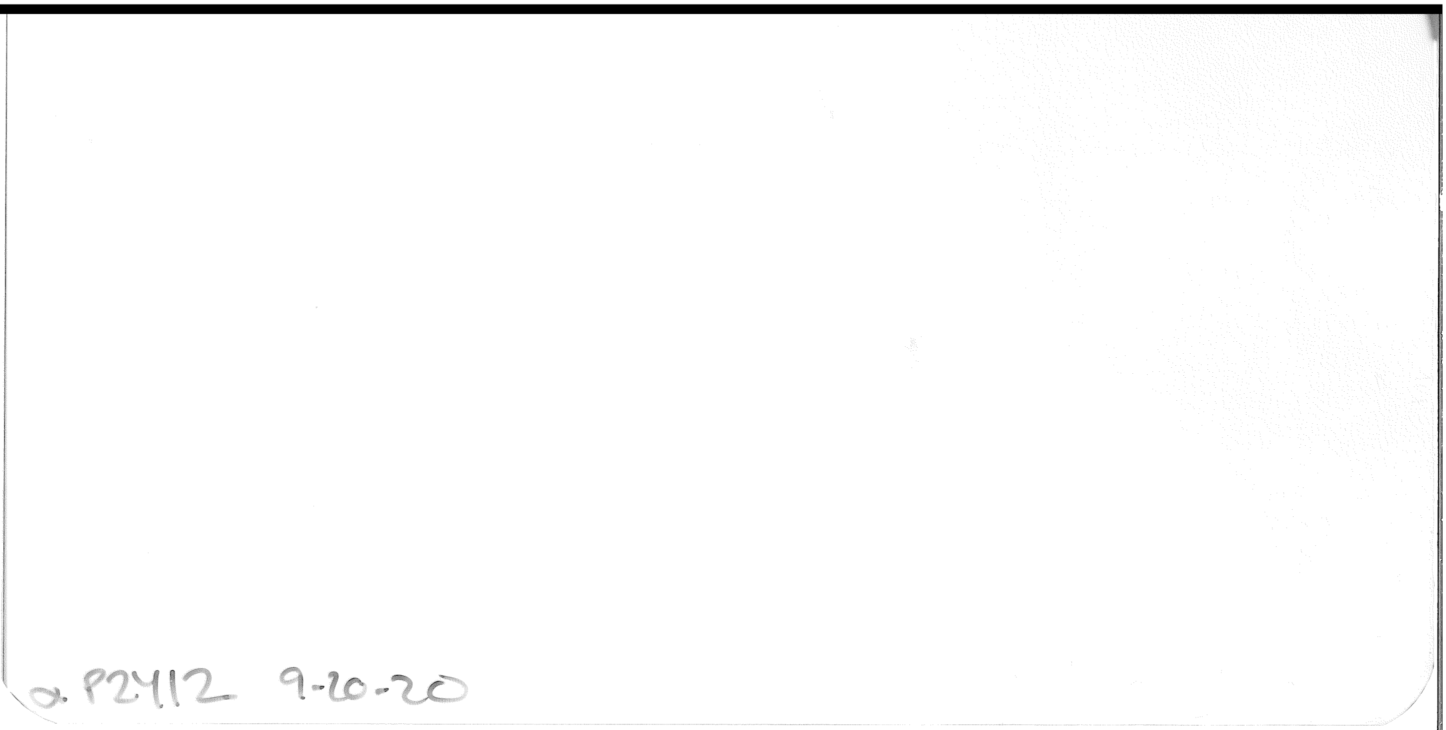

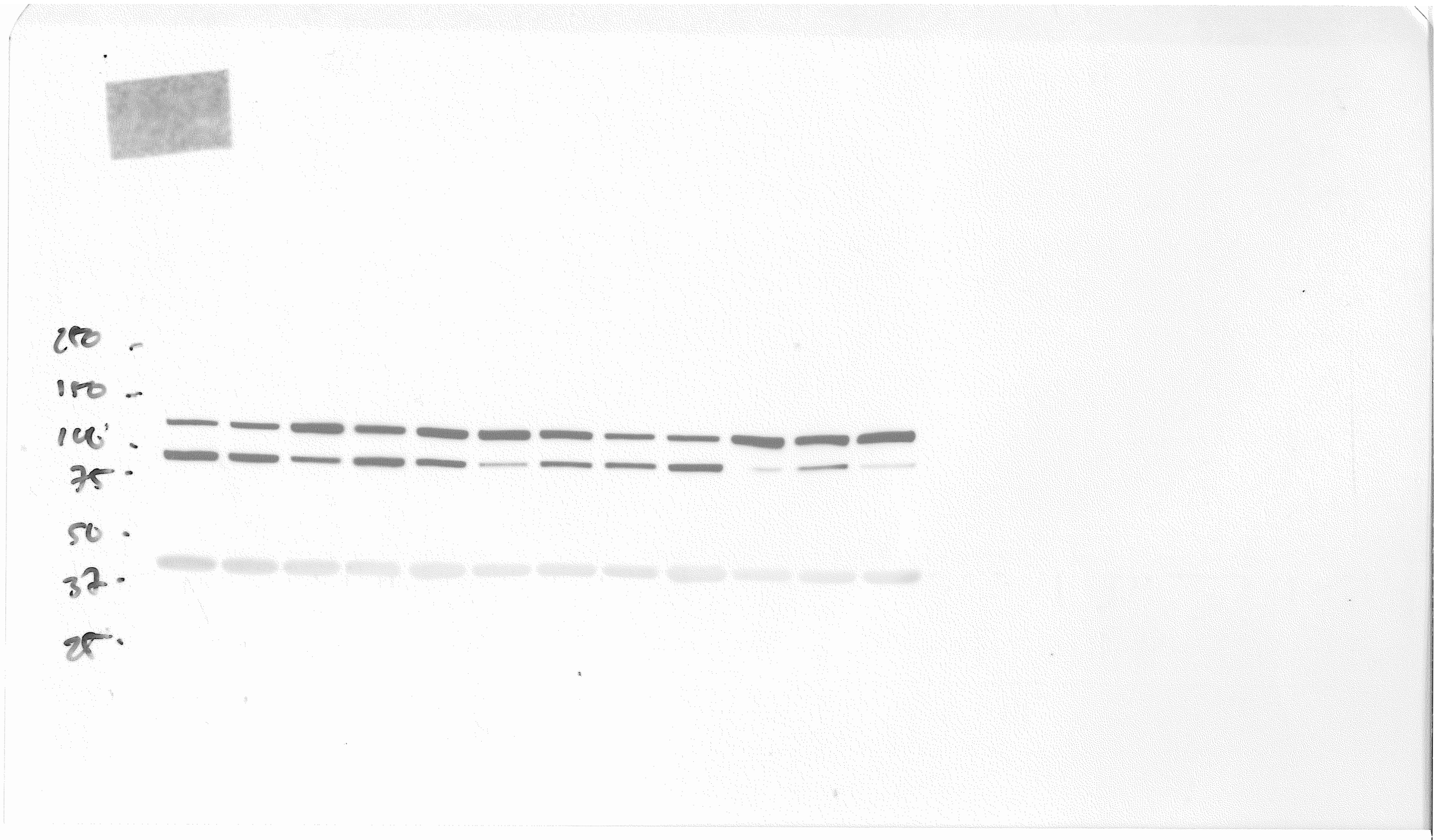


Full unedited gel for Figure 6D, depicting vinculin loading control at ~ 110 kda. Excess bands at ~100 kda and ~40 kda on the loading control membrane represent additional antibodies not included in the final version of this manuscript. Lanes 1-6 and 7-12 of the unedited gel correspond to those treatments shown in the cropped images within the manuscript: “Control” (Lanes 1-6) and “SCD” (Lanes 7-12).

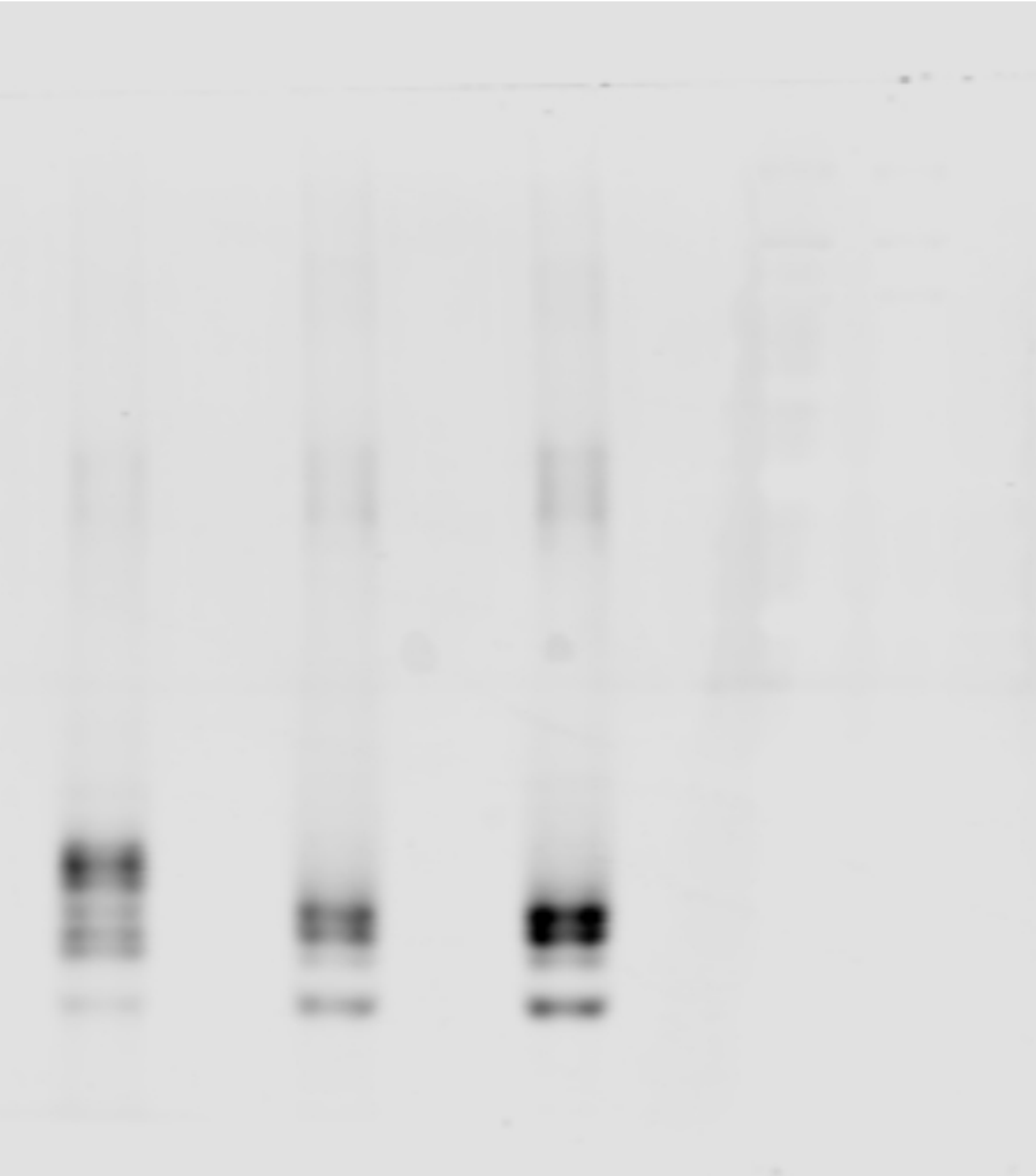

Full unedited gel for Supplemental Figure S1A, depicting biotin signal associated with 3 redox forms of recombinant HMGB1. Lanes 2, 4, and 6 of the unedited gel correspond to these treatments shown in the cropped images within the manuscript: “Untreated”, “Oxidized”, and “Reduced”. Lanes 8-9 contains the ladder and is not visible on the 800 channel. Blank lanes were left between loaded lanes containing sample.

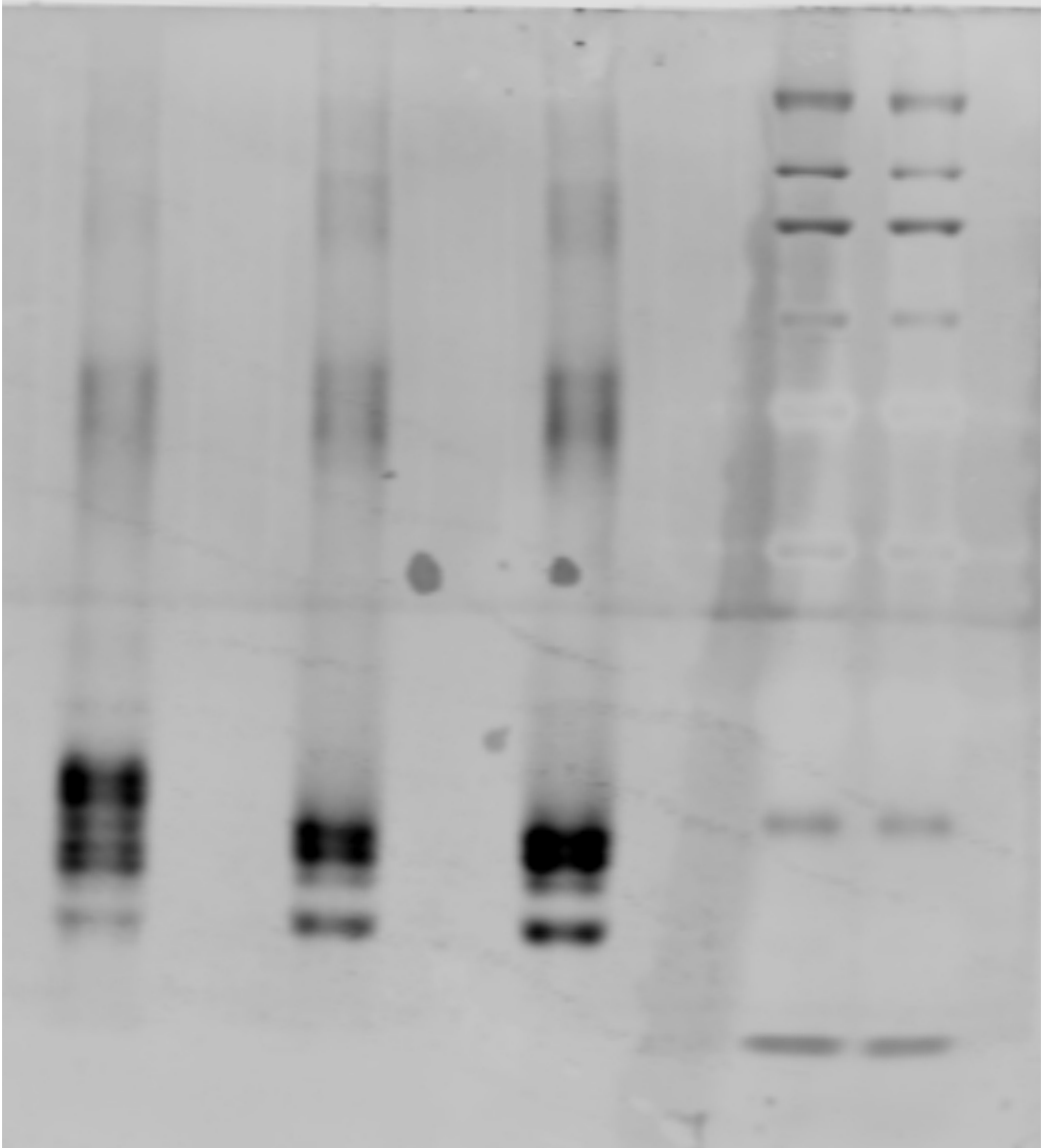

Full unedited gel for Supplemental Figure S1A, depicting HMGB1 signal. Lanes 2, 4, and 6 of the unedited gel correspond to those treatments shown in the cropped images withing the manuscript: “Untreated”, “Oxidized”, and “Reduced”. Blank lanes were left between loaded lanes containing sample.
